# Design, Synthesis and Fungicidal Activity of 2-Substituted Phenyl-2-oxo-, 2-Hydroxy- and 2-Acyloxyethylsulfonamides

**DOI:** 10.3390/molecules22050738

**Published:** 2017-05-04

**Authors:** Minlong Wang, Peng Rui, Caixiu Liu, Ying Du, Peiwen Qin, Zhiqiu Qi, Mingshan Ji, Xinghai Li, Zining Cui

**Affiliations:** 1 Department of Pesticide Science, Plant Protection College, Shenyang Agricultural University, Shenyang 110866, China; wangminlong0906@163.com (M.W.); ruiace@126.com (P.R.); LCX14714027@163.com (C.L.); duying92m@163.com (Y.D.); qinpeiwen08@sina.com (P.Q.); syqizhiqiu@sina.com (Z.Q.); jimingshan@163.com (M.J.); 2 State Key Laboratory for Conservation and Utilization of Subtropical Agro-Bioresources, Integrative Microbiology Research Centre, Guangdong Province Key Laboratory of Microbial Signals and Disease Control, South China Agricultural University, Guangzhou 510642, China; 3 Key Laboratory of Green Pesticide and Agricultural Bioengineering, Ministry of Education, Guizhou University, Guiyang 550025, China

**Keywords:** acetophenone, ethylsulfonamides, synthesis, fungicidal activity, *Botrytis cinerea*, structure-activity relationship

## Abstract

Sulfonyl-containing compounds, which exhibit a broad spectrum of biological activities, comprise a substantial proportion of and play a vital role, not only in medicines but also in agrochemicals. As a result increasing attention has been paid to the research and development of sulfonyl derivatives. A series of thirty-eight 2-substituted phenyl-2-oxo- **III**, 2-hydroxy- **IV** and 2-acyloxyethylsulfonamides **V** were obtained and their structures confirmed by IR, ^1^H-NMR, and elemental analysis. The in vitro and in vivo bioactivities against two *Botrytis cinerea* strains, **DL-11** and **HLD-15**, which differ in their sensitivity to procymidone, were evaluated. The in vitro activity results showed that the EC_50_ values of compounds **V-1** and **V-9** were 0.10, 0.01 mg L^−1^ against the sensitive strain **DL-11** and 3.32, 7.72 mg L^−1^ against the resistant strain **HLD-15**, respectively. For in vivo activity against *B. cinerea*, compound **V-13** and **V-14** showed better control effect than the commercial fungicides procymidone and pyrimethanil. The further in vitro bioassay showed that compounds **III**, **IV** and **V** had broad fungicidal spectra against different phytopathogenic fungi. Most of the title compounds showed high fungicidal activities, which could be used as lead compounds for further developing novel fungicidal compounds against *Botrytis cinerea*.

## 1. Introduction

Sulfonyl-containing compounds, which exhibit a broad spectrum of biological activities, comprise a substantial proportion of and play an important role not only in medicines but also in agrochemicals [1,2,3,4,5]. The sulfonyl group is widely applied in the field of functional organic molecule design due to its stable structure and high polarity with strong electron withdrawing ability. Sulfonyl groups can supply two hydrogen-bond acceptors, and a monosubstituted sulfonamide can supply an additional hydrogen-bond donor [6]. Structurally, the sulfonyl group possesses similar molecular size and charge distribution properties as carbonyl, carboxyl and phosphate groups, which can be replaced by a sulfonyl group as a bioisostere to retain or improve bioactivity [7,8,9]. In addition, the introduction of a sulfonyl group can also modulate the solubility and acid-base property of the functional molecules [10,11]. Since the sulfonyl group contains two hydrogen-bond acceptor centers, reasonable introduction of sulfonyl group can enhance the binding affinity of functional molecules with their target proteins to improve activity through hydrogen bond interactions [12,13]. Moreover, the introduction of a sulfonyl group can increase the metabolic stability of functional molecules to prolong the duration of action by blocking metabolically labile sites and increase the bioavailability [14,15,16]. Therefore, the development of sulfonyl-containing compounds has attracted considerable attention.

Sulfonamides were the first drugs with a selective effect on bacteria that could be used systemically against bacterial infections [17]. Hence, great attention has increasingly been paid to developing compounds containing sulfonyl groups as drugs and agrochemicals [18,19,20]. Sulfonamide fungicides, such as tolnifanide, cyazofamid and amisulbrom, have been commercialized, however, there are less sulfonamide species fungicides in field applications, as the advantages and action characteristics of these fungicides are sometimes not obvious in the field. Therefore, there is an urgent need to develop novel sulfonamide fungicides with improved properties [21]. Recently, a series of novel sulfonyl-containing compounds with different scaffolds, such as cycloalkylsulfonamide, benzenesulfonamide and their derivatives containing 1,3,4-thiadiazoles, coumarins and pyrans were reported for obvious and diverse fungicidal activity against *Phomopsis asparagi*, *Cladosporium fulvum* and *Fusarium oxysporum*, etc. [22,23,24,25,26]. The study of cycloalkylsulfonamides started from 2-oxocyclo-dodecylsulfonamide which showed good inhibitory activity against *Venturia nashicola* and *Fusarium graminearum* [27]. Using it as a lead compound, 2-oxocycloalkylsulfonamides were further investigated, and the novel candidate fungicide chesulfamide (codename CAUWL-2004-L-13) [28] has been developed to control phytopathogenic fungi including *Botrytis cinerea* and *Corynespora cassiicola*.

In our previous work, different scaffolds were introduced onto lead 2-oxocycloalkyl-sulfonamides, and the structure-activity relationships (SAR) were studied [29,30,31,32]. According to the results and following a program of extension and change of these compounds’ structures, we changed the naphthenic group linked with the sulfur bond in the sulfonamide group into an alkyl chain and found that the ethylsulfonamide group was the essential bioactive moiety and therefore, the ethylsulfonamide group was identified as a key building block for compound activity. Meanwhile, some benzoylmethanesulfonamide which showed better fungicidal activity than the cycloalkylsulfonamides were also synthesized [33]. Based on these results, 105 kinds of *N*-substituted-2-oxo-2-phenylethylsulfonamide derivatives were synthesized using combinatorial chemistry, and the fungicidal activity against *B. cinerea* were evaluated. Therefore, the SAR of substituent groups on the N atom such as monosubstituted anilines, multi-substituted anilines, substituted benzylamines and alkylamines were systematically studied [34].

**Figure 1:**
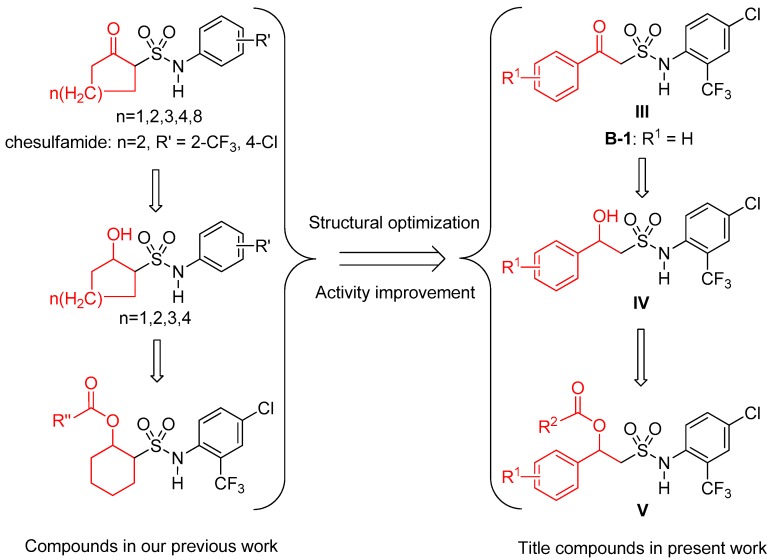
Design strategy of the title compounds.

**Scheme 1:**
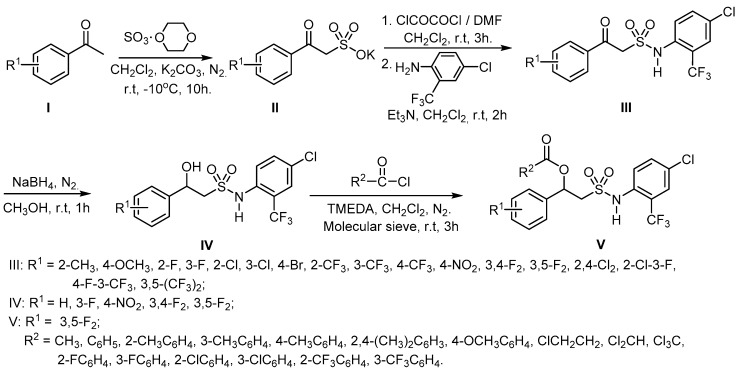
Synthesis of compounds **III**, **IV** and **V**.

On the basis of the above, in order to explore novel structure compounds with high fungicidal activity, and according to our SAR study, we designed and developed a series of new 2-substituted phenyl-2-oxo- **III**, 2-hydroxy- **IV** and 2-acyloxyethylsulfonamides **V** (Figure 1 and Scheme 1). The in vitro and in vivo activities against different strains of *Botrytis cinerea* were tested, and the fungicidal spectra of the title compounds were also determined.

## 2. Results

### 2.1. Chemistry

Potassium 2-substituted-phenyl-2-oxoethylsulfonates **II**, prepared from commercially available substituted acetophenones **I** by sulfonation with a sulfur trioxide-dioxane adduct and neutralized with potassium bicarbonate, were reacted with oxalyl chloride to give the corresponding 2-substituted-phenyl-2-oxoethylsulfonyl chlorides, which were converted into the title compounds **III** by amination with 2-trifluoromethyl-4-chlorophenylamine using triethylamine (Et_3_N) as catalyst.

According to the structure-activity relationships between substituents on the phenyl ring and fungicidal activity, some of these *N*-(2-trifluoromethyl-4-chlorophenyl)-2-substituted-phenyl-2-oxoethylsulfonamides **III** with high fungicidal activity were selected for further reaction with sodium borohydride, giving *N*-(2-trifluoromethyl-4-chlorophenyl)-2-hydroxy-2-substituted-phenylethyl-sulfonamides **IV**. Among these compounds *N*-(2-trifluoromethyl-4-chlorophenyl)-2-hydroxy-2-(3,5-difluorophenyl)ethylsulfonamide (**IV-5**) showed excellent fungicidal activity. Therefore, we further synthesized a series of *N*-(2-trifluoromethyl-4-chlorophenyl)-2-acyloxy-2-(3,5-difluorophenyl)ethyl-sulfonamides **V** by the reaction of **IV-5** with acyl chlorides in the presence of TMEDA and molecular sieves as catalysts. Finally, a total of 38 2-substituted-phenyl-2-oxo-, 2-hydroxy- and 2-acyloxy- ethylsulfonamides were synthesized. Flash chromatography was used for separation and purification and the structures of all the title compounds were confirmed by IR, ^1^H-NMR, and elemental analysis. The ^1^H-NMR spectra is available in Supplementary materials.

### 2.2. Biological Assay

The fungicidal activities of all the target compounds against two different strains of *Botrytis cinerea* with different sensitivity to procymidone collected from different areas in Liaoning, China were tested by in vitro mycelium growth inhibition assay and in vivo greenhouse pot experiments. Moreover, the fungicidal spectra against seven phytopathogenic fungi (*Pyricularia grisea*, *Exserohilum turcicum* (Pass.) Leonard et Suggs, *Pythium aphanidermatum*, *Phytophthora capsici* Leonian, *Fusarium graminearum* Schw., *Corynespora cassiicola* and *Thanatephorus cucumeris*) were also evaluated.

#### 2.2.1. In Vitro Fungicidal Activity against *Botrytis cinerea*

The fungicidal activities of *N*-(2-trifluoromethyl-4-chlorophenyl)-2-substituted-phenyl-2-oxoethylsulfonamides **III** against two different *B. cinerea* strains **DL-11** (a sensitive strain) and **HLD-15** (a resistant strain) were evaluated by an in vitro mycelium growth inhibition assay, and the commercial fungicides procymidone, chlorothalonil and pyrimethanil were used as the positive controls. The synthetic compound (*N*-(2-trifluoromethyl-4-chlorophenyl)-2-phenyl-2-oxoethyl-sulfonamide (**B-1**) was also used as the positive control to compare the difference between the pre- and post-structure optimization. The inhibition of mycelial growth was evaluated by measuring colony diameters in the presence and absence of the tested compounds. The results of preliminary bioactivity screening, expressed as the percentage of inhibition, showed that most of the compounds **III** showed high inhibition rate (more than 70% at 50 mg L^−1^) and better activity than that of **B-1**. Based on the experimental results, the EC_50_ values of the compounds **III** were next evaluated to confirm their fungicidal effects on the two different kinds of *B. cinerea* strains **DL-11** and **HLD-15**. The values are listed in Table 1.

As shown in Table 1, the EC_50_ values of compounds **III** against *B. cinerea* strains **DL-11** and **HLD-15** were within 1.58–10.81 mg L^−1^ and 3.48–17.70 mg L^−1^, respectively. Among them, compounds **III-13**, with EC_50_ values of 1.58 mg L^−1^ and 3.48 mg L^−1^ against **DL-11** and **HLD-15**, respectively, exhibited the best fungicidal activity against *B. cinerea*. The commercial fungicide procymidone showed EC_50_ values of 2.59 mg L^−1^ and 15.95 mg L^−1^, chlorothalonil gave EC_50_ values of 1.66 mg L^−1^ and 17.52 mg L^−1^ and pyrimethanil provided EC_50_ values of 32.73 mg L^−1^ and 71.25 mg L^−1^ against the two strains **DL-11** and **HLD-15,** against which **B-1**, another positive control, provided EC_50_ values of 14.25 mg L^−1^ and 22.79 mg L^−1^. The antifungal activity of compounds **III-8**, **III-14** and **III-15** was close to that of the control compound procymidone. With procymidone as a standard fungicide against *B. cinerea* strain **DL-11**, the relative activities of compounds **III-13** and **III-15** were 1.64- and 1.57-fold higher, respectively; with **B-1** as a standard fungicide, the relative activities of compounds **III-13** and **III-15** were 9.02- and 8.69 fold better, respectively. As regards to the control efficiency of *B. cinerea* strain **HLD-15**, compounds **III-13** and **III-15**, with 14.42 and 13.89 times higher activity than that of **B-1**, were 10.09 and 9.72 times better than procymidone, respectively.

The fungicidal activity of *N*-(2-trifluoromethyl-4-chlorophenyl)-2-hydroxy-2-substituted-phenyl ethylsulfonamides **IV** and *N*-(2-trifluoromethyl-4-chlorophenyl)-2-acyloxy-2-(3,5-difluoro-phenyl)ethylsulfonamides **V** against *B. cinerea* was also evaluated and the EC_50_ values are summarized in Table 1. The EC_50_ values of compounds **IV** against *B. cinerea* strains **DL-11** and **HLD-15** were within 0.70–13.33 mg L^−1^ and 0.61–33.16 mg L^−1^, respectively, and with compounds **V**, within 0.01–1279.16 mg L^−1^ and 3.32–3648.50 mg L^−1^, respectively. Some of compounds **IV** and **V** which were derived from compounds **III** showed better activity against *B. cinerea*. Compound **V-9** showed the best in vitro fungicidal activity against **DL-11**, with an EC_50_ value of 0.01 mg L^−1^, and **IV-5**, with an EC_50_ value of 0.61 mg L^−1^, exhibited the best activity against **HLD-15** in vitro. With procymidone as a standard fungicide against the *B. cinerea* strain **DL-11**, the relative activities of compounds **V-1** and **V-9** were 25.9- and 259-fold higher, respectively. As regards to the control effect of the strain **HLD-15**, compounds **IV-5** and **V-1** were 26.15- and 4.8-times more active than procymidone, respectively. Additionally, there is significantly different in the activity level among compounds **V**. For instance, compound **V-1** showed an EC_50_ values of 0.1 mg L^−1^ against the *B. cinerea* strain **DL-11**, but compound **V-6** showed an EC_50_ value of 131.12 mg L^−1^.

Finally, it was important to stress that most compounds **III** showed better fungicidal activity against *B. cinerea* strain **DL-11** (sensitive strain) than that against **HLD-15** (resistant strain) because of the resistance, but some individual compounds, on the contrary, exhibited better activity against **HLD-15**. In general, compounds should exhibit better activity against sensitive strains than resistant strains. Moreover, the same compound gave different control effects on the different *B. cinerea* strains owing to the difference of biological characteristics, pathogenicity and resistant to fungicides, but on the whole, there were consistent trends in the control effects against both **DL-11** and **HLD-15**.

#### 2.2.2. In Vivo Fungicidal Activity against *Botrytis cinerea*

The in vivo fungicidal activity of compounds **III**, **IV** and **V** against *B. cinerea* was evaluated and the results listed in Table 2. The results showed that compounds **III**, **IV** and **V** showed good inhibition, and some of them exhibited better control than the commercial fungicides procymidone and pyrimethanil. Among them, compounds **V-13** demonstrated the best activity, with a control efficiency of 74.6%. Besides, the control efficiencies of **V-1**, **V-6**, **V-8**, **V-10**, **V-13** and **V-14** were 70.8%, 71.3%, 70.8%, 71.3%, 74.6% and 73.3%, respectively, which were similar to that of the commercial fungicide procymidone (with a control efficiency of 72.5%).

The tested results showed that the whole level of the control effects of compounds **IV** and **V** was somewhat increased in comparison to compounds **III**, especially compounds **V**, all of which showed control efficiencies of more than 60%. In addition, the in vitro fungicidal activity of the tested compounds showed no consistent correlation with that in vivo. Compound **V-13**, with the high EC_50_ values of 35.18 mg L^−1^ in vitro, had the highest control efficiency of 74.6% against the *B. cinerea* strain **DL-11**. The reason may be that, there were interactions between reagents and plants in vivo experiments, not just between the reagents and the fungi. Therefore, characteristics of the tested compounds such as polarity, conductibility and degradation in vivo also had influence on the fungicidal activity, which is worthy of further study.

#### 2.2.3. Fungicidal Activity against Different Phytopathogenic Fungi

Different kinds of phytopathogenic fungi were chosen to evaluate the fungicidal spectrum of the title compounds **III**, **IV** and **V**. We summarize the bioassay results of inhibitory activities of these compounds against seven phytopathogenic fungi at 50 mg L^−1^. Overall, the title compounds showed different levels of fungicidal activity. Compounds **III-2**, **III-10**, **III-16**, **IV-5** and **V-10** exhibited wide spectrum antifungal activity and compound **V-10** displayed excellent activities against all the tested phytopathogentic fungi. Moreover, some compounds showed highly specific pathogen activity, whereby compounds **III-1** and **III-4** exhibited activities against *Phytophthora capsici* Leonian with inhibition rates 90.4% and 91.0%, respectively. It is noteworthy that nearly all compounds **III** showed excellent fungicidal activity against *Phytophthora capsici* Leonian and *Fusarium graminearum* Schw., and most of the inhibition rate was more than 70%. In general, compounds **V** possessed an improvement of fungicidal activity in comparison to compounds **III**. However, compounds **V** were only slightly active against *Exserohilum turcicum* (Pass.) Leonard et Suggs and *Pythium aphanidermatum*, being less active than compounds **III**. Besides, some individual compound showed unexpected antifungal activity against a certain pathogen. For example, Compound **V-16** exhibited good activity against *P. aphanidermatum* with inhibition rate of 72.9%, although the inhibition rates of most compounds **V** were less than 25%.

Based on the inhibition rate of compounds **III**, **IV** and **V** against different phytopathogentic fungi, EC_50_ values of some highly bioactive compounds were evaluated against four different phytopathogentic fungi (*Fusarium graminearum* Schw., *Thanatephorus cucumeris*, *Pythium aphanidermatum* and *Phytophthora capsici* Leonian). Compound **B-1** and chlorothalonil were used as the positive controls. As shown in Table 3, compounds **III-6**, **III-10**, **III-13** and **V-10** exhibited the best fungicidal activity against the four fungi, with EC_50_ values of 1.41, 0.99, 7.25, 2.25 mg L^−1^; 1.05, 0.60, 8.65, 4.47 mg L^−1^; 0.79, 1.86, 8.62, 2.74 mg L^−1^ and 2.41, 1.07, 6.96, 3.22 mg L^−1^, respectively.

## 3. Discussion

### 3.1. Synthesis and Structure Elucidation

High yield syntheses of compounds **III**, **IV** and **V** were accomplished in short reaction times and using mild reaction conditions. Oleum was often used as an efficient sulfonating agent, but lots of byproducts are generated during the sulphonation process with that reagent. In our reactions, sulfur trioxide-dioxane adduct was used as the sulfonating agent, prepared by a standard procedure (mixing a solution of sulfur trioxide in 1,2-dichloroethane with a solution of dioxane in the same solvent in a certain order to give 1:1 sulfur trioxide-dioxane adducts), and high yields of products were achieved. Besides, we also tried to react sulfur trioxide-pyridine adduct with the acetophenones, but the yields of this reaction were very low, and in some cases no product was obtained. A tentative inference from this result was that the relative stability and reactivity of sulfur trioxide-pyridine adduct and sulfur trioxide-dioxane adduct has an influence on the sulfonation of acetophenones. Moreover, oxalyl chloride was used as the chlorinating agent in these experiments to give the corresponding sulfonyl chlorides and acyl chlorides, which were reacted with DMF catalysis. The reaction proceeded smoothly in high yields and the product separation and purification were simple. In most cases, the title compounds **III**, **IV** and **V** were obtained in acceptable yield.

**Figure 2:**
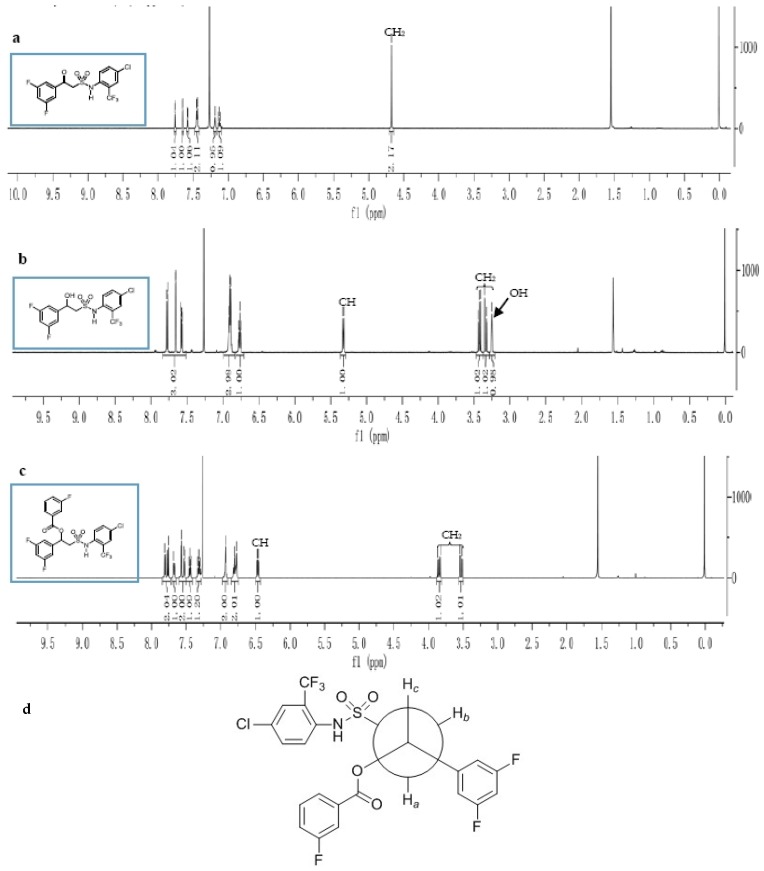
Structure elucidation of compounds **III**, **IV** and **V**: (**a**) ^1^H-NMR spectrum of compound **III-13**; (**b**) ^1^H-NMR spectrum of the compound **IV-5**; (**c**) ^1^H-NMR spectrum of compound **V-12**; (**d**) The proposed stable conformation of compound **V-12**.

The chemical structures of all the synthesized compounds in this work were mainly characterized by nuclear magnetic resonance (NMR) and infrared (IR) absorption spectroscopy. The ^1^H-NMR spectra of *N*-(2-trifluoromethyl-4-chlorophenyl)-2-substituted-phenyl-2-oxoethyl-sulfonamides **III** showed some characteristic peaks, i.e., the ^1^H chemical shifts of the phenyl rings in the sulfonamides were seen at around 7.50 ppm. The ^1^H-NMR spectrum of compounds **III** showed a singlet signal around 4.70 ppm, which was assigned to the methylene protons between the sulfonyl and carbonyl groups. Additionally, a signal appearing at approximately 7.25 ppm was assigned to the NH group in the sulfonamide functionality (Figure 2a).

In the IR spectra, a carbonyl stretching vibration appeared, as expected, around 1680 cm^−1^ and 1700 cm^−1^. The NH stretching vibration was around 3250 cm^−1^ to 3350 cm^−1^. The spectra of compounds **IV**, which were obtained in high yield (more than 85%) by reduction of compounds **III**, showed significant changes compared with those of compounds **III**. The signal of the methylene group in the ^1^H-NMR spectra appeared as a multiplet due to the effect of the chiral carbon in the side chain. Therefore, the ^1^H-NMR chemical shifts of the CH_2_ combined with the sulfonyl and CH linked with the hydroxyl group were at about 3.40 ppm and 5.30 ppm, respectively. A signal appearing at approximately 3.20 ppm was assigned to the OH group (Figure 2b). In the IR spectrum, it was found that carbonyl absorption peak at 1680 cm^−1^ disappeared and that of a hydroxyl with an obvious stretching vibration at 3400 cm^−1^ appeared. For compounds **V**, the ester carbonyl stretching vibration appeared around 1700 cm^−1^. In addition, the most stable conformation of compound **V-12** (Figure 2c,d) could be proposed. As shown in Figure 2d, the two hydrogens (H_a_, H_b_) of methylene were of chemical unequivalent. The ^1^H-NMR analysis showed that the coupling constant of H_a_ and H_b_ was ^1^*J* = 7.35 Hz, and that of H_a_ and H_c_, H_b_ and H_c_ were ^2^*J* = 1.56 Hz, ^3^*J* = 4.83 Hz, respectively (Figure 2c).

### 3.2. Screening of Fungicidal Activity and Structure-Activity Relationships

In this paper, the fungicidal activities of compounds **III**, **IV** and **V** against two *Botrytis cinerea* strains collected from different areas in Liaoning, China were tested by an in vitro mycelium growth inhibition assay and in vivo greenhouse pot experiments. By adopting different populations of the same species, the analytical activity screening results were more accurate and reliable, and then this made the activity evaluation of synthetic compounds against fungi more comprehensive and persuasive. Comprehensively, the fungicidal activities of the same compound against different *Botrytis cinerea* strains showed less differences owing to the difference of biological characteristics, pathogenicity and resistance to fungicides between isolates. Hence, certain compounds showed excellent antifungal activity against a certain strain, whereas for another isolate, the activity may be unsatisfactory. For the purpose of screening new compounds worthy of further research, we selected compounds with excellent fungicidal activities against all the tested *Botrytis cinerea* strains, especially for the resistant strain. Thus, compounds such as **III-12**, **III-13** were selected for further activity screening and structure optimization.

From the results of the fungicidal activity screening against *Botrytis cinerea* and the structure-activity relationships (SAR), it could be seen that the substituents on the benzene ring might exert a greater influence on the activity. As for *N*-(2-trifluoromethyl-4-chlorophenyl)-2-substituted-phenyl-2-oxoethylsulfonamides **III**, the biological experiments showed that the introduction of any substituent in the phenyl ring increased the inhibition activity to *Botrytis cinerea*. The activity was significantly increased when the benzene ring contained fluorine-containing groups (F and CF_3_) which show significant functions in organisms due to their osmotic and electronic effects, etc. The number of fluorine-containing groups also influenced the antifungal activity. Thus, when the substituent was fluorine, the fungicidal activity increased with the increase of the number of substituents. If the substituent was a trifluoromethyl group, contrarily the fungicidal activity decreased. Moreover, there was no significant difference between different positions of halogen atoms on the benzene ring and their fungicidal activities. However, for trifluoromethyl, it seemed that the compound with a trifluoromethyl group on the *para* position of the benzene ring showed higher activities than when it was at the *meta* position, and the compound with a trifluoromethyl group on the *para* position exhibited the best activity. We also revealed that the strong electron-donating capacity of the substituent on the phenyl ring reduced the fungicidal activity of compounds **III**. For example, compounds **III-1** and **III-3** (with the EC_50_ values of 8.67, and 7.51 mg L^−1^ against the *Botrytis cinerea* strain **DL-11**, respectively) had lower fungicidal activity than the compound **III-13** (with an EC_50_ value of 1.58 mg L^−1^ against the *Botrytis cinerea* strain **DL-11**). The structure-activity relationship of *N*-(2-trifluoromethyl-4-chlorophenyl)-2-hydroxy-2-substituted-phenyl ethyl-sulfonamides **IV** was similar to that of compounds **III**, while for *N*-(2-trifluoromethyl-4-chlorophenyl)-2-acyloxy-2-(3,5-difluorophenyl)ethylsulfonamides **V**, it could be seen that the acyloxy substituent showed higher fungicidal activity when it was a small group. The compounds with an alkylacyloxy groups showed higher activities than that with an arylacyloxy group, and generally substituted benzoyloxy compounds showed lower activity than benzoyloxy compounds. For instance, compounds **V-1**, **V-10** and **V-15** provided EC_50_ values of 0.10, 1.18 and 20.33 mg L^−1^ against the *Botrytis cinerea* strain **DL-11**, respectively. The fungicidal activities showed no significant difference between compounds **V** with the change of substituent position of the substituted benzoyloxy group.

It is worth mentioning that the fungicidal activities showed no significant difference between compounds **III** and **IV**, most of which had inhibition rates of more than 75.0% against *Botrytis cinerea*, although for compounds **V**, there were obvious differences between their individual fungicidal activities. This suggests that these compounds have different modes of action, which need to be further studied. Additionally, the results of in vitro and in vivo fungicidal activities were inconsistent. For example, with procymidone as a standard fungicide against *Botrytis cinerea* strains **DL-11**, the relative toxicity of compound **V-9** was 259, which represented the best activity in vitro. However, the in vivo activity of **V-10** only showed the control efficiency of 65.4%, and the positive control procymidone had a control efficiency of 58.8%. Further investigation are needed to explain these findings.

## 4. Materials and Methods

### 4.1. General Information

The melting points were measured using an X-5 melting-point apparatus (Beijing Second Optical Instrument Factory, Beijing, China). Infrared (IR) spectra were recorded in potassium bromide disks on a Spectrum 65 spectrophotometer (Perkin Elmer, Waltham, MA, USA). Nuclear magnetic resonance (NMR) spectra were recorded in CDCl_3_ on Bruker 400 or 600 MHz spectrometers (Bruker, Shanghai, China), using tetramethylsilane (TMS) as an internal standard. Elemental analyses were determined on a Vario EL III elemental analyser (Elementar Analysensysteme GmbH, Frankfurt, Germany).

### 4.2. Synthetic Procedures

The synthetic route of the title compounds is shown in Scheme 1. Potassium 2-substituted phenyl-2-oxoethylsulfonates **II**, were prepared from readily commercially available substituted acetophenones **I** by sulfonation with a sulfur trioxide-dioxane adduct and neutralization with potassium carbonate according to the method given in references [27,29,30]. The title compounds were synthesized by a method developed by our group described as follows:

#### 4.2.1. General Synthetic Procedure for the Target Compounds **III**

To a slurry of potassium 2-substituted0phenyl-2-oxoethylsulfonate **II** (0.03 mol) and *N*,*N*-dimethylformamide (DMF, 0.15 mL) in dichloromethane (CH_2_Cl_2_, 30 mL), oxalyl chloride (0.033 mol) was added dropwise at −5 °C. The mixture was stirred at room temperature for 3 h. After cooling in an ice-water bath, it was filtered under reduced pressure. The filtrate was added dropwise to a solution of 2-amino-5-chlorobenzotrifluoride (0.015 mol) and triethylamine (Et_3_N, 0.03 mol) in CH_2_Cl_2_ (20 mL) at −5 °C. After stirring at room temperature for 3 h, the reaction mixture was washed successively with 3 mol L^−1^ hydrochloric acid (HCl, 15 mL), saturated sodium bicarbonate solution (15 mL) and saturated salt water (15 mL), and then dried over sodium sulfate. After evaporating the solvent under vacuum, the crude product was further purified by silica column chromatography to give pure compounds **III-1** to **III-17** using the mixture of ethyl acetate and petroleum ether (10:1) as the eluent.

*N-(2-Trifluoromethyl-4-chlorophenyl)-2-(2-methylphenyl)-2-oxoethylsulfonamide* (**III-1**): white solid, 88.1% yield: m.p. 59–61 °C; IR (KBr) ν_max_ 3255, 3045, 2917, 1689, 1338, 1130 cm^−1^; ^1^H-NMR (400 MHz), δ (ppm) 2.25 (s, 3H, CH_3_), 4.71 (s, 2H, CH_2_), 7.37 (S, 1H, NH), 7.29–7.75 (m, 7H, C_6_H_4_ + C_6_H_3_); Elemental anal. calc. for C_16_H_13_ClF_3_NO_3_S (%): C, 49.05; H, 3.34; N, 3.58; found: C, 49.33; H, 3.20; N, 3.78.

*N-(2-Trifluoromethyl-4-chlorophenyl)-2-(4-methoxylphenyl)-2-oxoethylsulfonamide* (**III-2**): light brown solid, 83.6% yield: m.p. 83–85 °C; IR (KBr) ν_max_ 3350, 3080, 2968, 1670, 1344, 1161 cm^−1^; ^1^H-NMR (400 MHz), δ (ppm) 3.90 (s, 3H, CH_3_), 4.71 (s, 2H, CH_2_), 7.37 (S, 1H, NH), 6.97–7.92 (m, 7H, C_6_H_3_ + C_6_H_4_); Elemental anal. calc. for C_16_H_13_ClF_3_NO_4_S (%): C, 47.13; H, 3.21; N, 3.43; found: C, 47.36; H, 3.48; N, 3.66.

*N-(2-Trifluoromethyl-4-chlorophenyl)-2-(2-fluorophenyl)-2-oxoethylsulfonamide* (**III-3**): white solid, 78.9% yield: m.p. 122–124 °C; IR (KBr) ν_max_ 3296, 3033, 2941, 1689, 1311, 1116 cm^−1^; ^1^H-NMR (400 MHz), δ (ppm) 4.78 (d, 2H, CH_2_), 7.17–7.20 (q, 1H, NH), 7.30–7.97 (m, 7H, C_6_H_4_ + C_6_H_3_); Elemental anal. calc. for C_15_H_10_ClF_4_NO_3_S (%): C, 45.52; H, 2.55; N, 3.54; found: C, 45.68; H, 2.34; N, 3.74.

*N-(2-Trifluoromethyl-4-chlorophenyl)-2-(3-fluorophenyl)-2-oxoethylsulfonamide* (**III-4**): light yellow solid, 81.5% yield: m.p. 109–111 °C; IR (KBr) ν_max_ 3342, 3078, 2974, 1687, 1311, 1122 cm^−1^; ^1^H-NMR (400 MHz), δ (ppm) 4.72 (s, 2H, CH_2_), 7.25 (s, 1H, NH), 7.35–7.77 (m, 7H, C_6_H_4_ + C_6_H_3_); Elemental anal. calc. for C_15_H_10_ClF_4_NO_3_S (%): C, 45.52; H, 2.55; N, 3.54; found: C, 45.36; H, 2.37; N, 3.68.

*N-(2-Trifluoromethyl-4-chlorophenyl)-2-(2-chlorophenyl)-2-oxoethylsulfonamide* (**III-5**): light yellow solid, 76.6% yield: m.p. 85–87 °C; IR (KBr) ν_max_ 3253, 3064, 2927, 1689, 1307, 1168 cm^−1^; ^1^H-NMR (400 MHz), δ (ppm) 4.84 (s, 2H, CH_2_), 7.25 (s, 1H, NH), 7.29–7.75 (m, 7H, C_6_H_4_ + C_6_H_3_); Elemental anal. calc. for C_15_H_10_Cl_2_F_3_NO_3_S (%): C, 43.71; H, 2.45; N, 3.40; found: C, 43.90; H, 2.59; N, 3.28.

*N-(2-Trifluoromethyl-4-chlorophenyl)-2-(3-chlorophenyl)-2-oxoethylsulfonamide* (**III-6**): light yellow solid, 68.4% yield: m.p. 109–111 °C; IR (KBr) ν_max_ 3340, 3056, 2974, 1687, 1338, 1116 cm^−1^; ^1^H-NMR (400 MHz), δ (ppm) 4.72 (s, 2H, CH_2_), 7.25 (s, 1H, NH), 7.45–7.91 (m, 7H, C_6_H_4_ + C_6_H_3_); Elemental anal. calc. for C_15_H_10_Cl_2_F_3_NO_3_S (%): C, 43.71; H, 2.45; N, 3.40; found: C, 44.01; H, 2.19; N, 3.25.

*N-(2-Trifluoromethyl-4-chlorophenyl)-2-(4-bromophenyl)-2-oxoethylsulfonamide* (**III-7**): light yellow solid, 78.9% yield: m.p. 89–91 °C; IR (KBr) ν_max_ 3292, 3033, 2926, 1687, 1396, 1120 cm^−1^; ^1^H-NMR (400 MHz), δ (ppm) 4.71 (s, 2H, CH_2_), 7.25 (s, 1H, NH), 7.55–7.81 (m, 7H, C_6_H_4_ + C_6_H_3_); Elemental anal. calc. for C_15_H_10_BrClF_3_NO_3_S (%): C, 39.45; H, 2.21; N, 3.07; found: C, 39.25; H, 2.03; N, 3.24.

*N-(2-Trifluoromethyl-4-chlorophenyl)-2-(2-trifluoromethylphenyl)-2-oxoethylsulfonamide* (**III-8**): light yellow solid, 74.3% yield: m.p. 79–81 °C; IR (KBr) ν_max_ 3298, 3032, 2943, 1705, 1315, 1114 cm^−1^; ^1^H-NMR (400 MHz), δ (ppm) 4.62 (s, 2H, CH_2_), 7.19 (s, 1H, NH), 7.55–7.78 (m, 7H, C_6_H_4_ + C_6_H_3_); Elemental anal. calc. for C_16_H_10_ClF_6_NO_3_S (%): C, 43.11; H, 2.26; N, 3.14; found: C, 43.35; H, 2.05; N, 3.36.

*N-(2-Trifluoromethyl-4-chlorophenyl)-2-(3-trifluoromethylphenyl)-2-oxoethylsulfonamide* (**III-9**): white solid, 77.6% yield: m.p. 85–87 °C; IR (KBr) ν_max_ 3348, 3027, 2972, 1689, 1338, 1184 cm^−1^; ^1^H-NMR (400 MHz), δ (ppm) 4.77 (s, 2H, CH_2_), 7.24 (s, 1H, NH), 7.55–8.18 (m, 7H, C_6_H_4_ + C_6_H_3_); Elemental anal. calc. for C_16_H_10_ClF_6_NO_3_S (%): C, 43.11; H, 2.26; N, 3.14; found: C, 43.31; H, 2.04; N, 2.99.

*N-(2-Trifluoromethyl-4-chlorophenyl)-2-(4-trifluoromethylphenyl)-2-oxoethylsulfonamide* (**III-10**): light yellow solid, 79.8% yield: m.p. 116–118 °C; IR (KBr) ν_max_ 3311, 3021, 2960, 1680, 1323, 1134 cm^−1^; ^1^H-NMR (400 MHz), δ (ppm) 4.75 (s, 2H, CH_2_), 7.22 (s, 1H, NH), 7.57–8.05 (m, 7H, C_6_H_4_ + C_6_H_3_); Elemental anal. calc. for C_16_H_10_ClF_6_NO_3_S (%): C, 43.11; H, 2.26; N, 3.14; found: C, 42.92; H, 2.50; N, 3.32.

*N-(2-Trifluoromethyl-4-chlorophenyl)-2-(4-nitrophenyl)-2-oxoethylsulfonamide* (**III-11**): light yellow solid, 70.5% yield: m.p. 119–121 °C; IR (KBr) ν_max_ 3273, 3010, 2954, 1703, 1396, 1122 cm^−1^; ^1^H-NMR (400 MHz), δ (ppm) 4.77 (s, 2H, CH_2_), 7.17 (s, 1H, NH), 7.58–8.39 (m, 7H, C_6_H_4_ + C_6_H_3_); Elemental anal. calc. for C_15_H_10_ClF_3_N_2_O_5_S (%): C, 42.62; H, 2.38; N, 6.63; found: C, 42.84; H, 2.18; N, 6.47.

*N-(2-Trifluoromethyl-4-chlorophenyl)-2-(3,4-difluorophenyl)-2-oxoethylsulfonamide* (**III-1**2): white solid, 71.5% yield: m.p. 89–91 °C; IR (KBr) ν_max_ 3356, 3078, 2972, 1687, 1313, 1176 cm^−1^; ^1^H-NMR (400 MHz), δ (ppm) 4.69 (s, 2H, CH_2_), 7.21 (s, 1H, NH), 7.30–7.82 (m, 6H, C_6_H_3_ + C_6_H_3_); Elemental anal. calc. for C_15_H_9_ClF_5_NO_3_S (%): C, 43.55; H, 2.19; N, 3.39; found: C, 43.82; H, 1.98; N, 3.54.

*N-(2-Trifluoromethyl-4-chlorophenyl)-2-(3,5-difluorophenyl)-2-oxoethylsulfonamide* (**III-13**): light yellow solid, 75.5% yield: m.p. 93–95 °C; IR (KBr) ν_max_ 3334, 3011, 2926, 1680, 1338, 1122 cm^−1^; ^1^H-NMR (400 MHz), δ (ppm) 4.67 (s, 2H, CH_2_), 7.19 (s, 1H, NH), 7.17–7.77 (m, 6H, C_6_H_3_ + C_6_H_3_); Elemental anal. calc. for C_15_H_9_ClF_5_NO_3_S (%): C, 43.55; H, 2.19; N, 3.39; found: C, 43.72; H, 2.35; N, 3.18.

*N-(2-Trifluoromethyl-4-chlorophenyl)-2-(2,4-dichlorophenyl)-2-oxoethylsulfonamide* (**III-14**): light yellow solid, 80.6% yield: m.p. 106–108 °C; IR (KBr) ν_max_ 3290, 3017, 2946, 1687, 1340, 1120 cm^−1^; ^1^H-NMR (CDCl_3_, 400 MHz), δ (ppm) 4.80 (s, 2H, CH_2_), 7.20 (s, 1H, NH), 7.38–7.75 (m, 6H, C_6_H_3_ + C_6_H_3_); Elemental anal. calc. for C_15_H_9_Cl_2_F_3_NO_3_S (%): C, 40.34; H, 2.03; N, 3.14; found: C, 40.58; H, 2.31; N, 3.32.

*N-(2-Trifluoromethyl-4-chlorophenyl)-2-(2-chloro-3-fluorophenyl)-2-oxoethylsulfonamide* (**III-15**): white solid, 72.8% yield: m.p. 115–117 °C; IR (KBr) ν_max_ 3309, 3014, 2916, 1730, 1338, 1122 cm^−1^; ^1^H-NMR (400 MHz), δ (ppm) 4.53 (s, 2H, CH_2_), 7.15(s, 1H, NH), 7.22–7.78 (m, 6H, C_6_H_3_ + C_6_H_3_); Elemental anal. calc. for C_15_H_9_Cl_2_F_4_NO_3_S (%): C, 41.88; H, 2.11; N, 3.26; found: C, 42.01; H, 1.97; N, 3.42.

*N-(2-Trifluoromethyl-4-chlorophenyl)-2-(4-fluoro-3-trifluoromethylphenyl)-2-oxoethylsulfonamide* (**III-16**): light yellow solid, 84.6% yield: m.p. 99–101 °C; IR (KBr) ν_max_ 3354, 3072, 2964, 1685, 1350, 1174 cm^−1^; ^1^H-NMR (400 MHz), δ (ppm) 4.73 (s, 2H, CH_2_), 7.20 (s, 1H, NH), 7.37–8.23 (m, 6H, C_6_H_3_ + C_6_H_3_); Elemental anal. calc. for C_16_H_9_ClF_7_NO_3_S (%): C, 41.44; H, 1.96; N, 3.02; found: C, 41.66; H, 2.10; N, 3.29.

*N-(2-Trifluoromethyl-4-chlorophenyl)-2-(3,5-ditrifluoromethylphenyl)-2-oxoethylsulfonamide* (**III-17**): light yellow solid, 69.7% yield: m.p. 119–121 °C; IR (KBr) ν_max_ 3354, 3072, 2964, 1685, 1350, 1174 cm^−1^; ^1^H-NMR (400 MHz), δ (ppm) 4.77 (s, 2H, CH_2_), 7.15 (s, 1H, NH), 7.57–8.35 (m, 6H, C_6_H_3_ + C_6_H_3_); Elemental anal. calc. for C_17_H_9_ClF_9_NO_3_S (%): C, 39.74; H, 1.77; N, 2.73; found: C, 39.55; H, 1.52; N, 2.99.

#### 4.2.2. General Synthetic Procedure for the Target Compounds **IV**

To a solution of *N*-(2-trifluoromethyl-4-chlorophenyl)-2-phenyl-2-oxoethylsulfonamide (0.01 mol) in methanol (30 mL) at 0–5 °C, sodium borohydride solution (0.016 mol NaBH_4_ + 8 mL of 1% NaOH + 8 mL of CH_3_OH) was added dropwise. The mixture was stirred at 5–25 °C for 1–3 h. After methanol was evaporated in vacuum, the residue was dissolved in ethyl acetate (80 mL), washed with 5% HCl (30 mL) and water (20 mL), and dried over sodium sulfate. After the solvent was evaporated under vacuum, the crude product was recrystallized from acetone/petroleum ether to give pure compounds **IV-1** to **IV-5**.

*N-(2-Trifluoromethyl-4-chlorophenyl)-2-phenyl-2-hydroxyethylsulfonamide* (**IV-1**): white solid, 87.2% yield: m.p. 95–96 °C; IR (KBr) ν_max_ 3242, 3061, 1369, 1315, 1128, 975; ^1^H-NMR (400 MHz), δ (ppm) 3.03 (s, 1H, OH), 3.37–3.67 (m, 2H, CH_2_), 5.35–5.36 (d, 1H, CH), 7.06–7.79 (m, 9H, C_6_H_5_ + C_6_H_3_ + NH); Elemental anal. calc. for C_15_H_13_ClF_3_NO_3_S (%): C, 47.44; H, 3.45; N, 3.69; found: C, 47.69; H, 3.56; N, 3.49.

*N-(2-Trifluoromethyl-4-chlorophenyl)-2-(3-fluorophenyl)-2-hydroxyethylsulfonamide* (**IV-2**): white solid, 88.6% yield: m.p. 78–80 °C; IR (KBr) ν_max_ 3241, 3024, 1368, 1316, 1134, 984; ^1^H-NMR (400 MHz), δ (ppm) 3.12 (s, 1H, OH), 3.36–3.54 (m, 2H, CH_2_), 5.35 (d, 1H, CH), 6.96–7.79 (m, 8H, C_6_H_4_ + C_6_H_3_ + NH); Elemental anal. calc. for C_15_H_12_ClF_4_NO_3_S (%): C, 45.29; H, 3.04; N, 3.52; found: C, 45.06; H, 3.21; N, 3.67.

*N-(2-Trifluoromethyl-4-chlorophenyl)-2-(4-nitrophenyl)-2-hydroxyethylsulfonamide* (**IV-3**): white solid, 94.1% yield: m.p. 110–111 °C; IR (KBr) ν_max_ 3256, 3054, 1385, 1320, 1122, 956; ^1^H-NMR (400 MHz), δ (ppm) 3.35–3.47 (m, 3H, CH_2_+OH), 5.45–5.47 (q, 1H, CH), 6.91–8.25 (m, 8H, C_6_H_4_ + C_6_H_3_ + NH); Elemental anal. calc. for C_15_H_12_ClF_3_N_2_O_5_S (%): C, 42.41; H, 2.85; N, 6.60; found: C, 42.58; H, 3.01; N, 6.48.

*N-(2-Trifluoromethyl-4-chlorophenyl)-2-(3,4-difluorophenyl)-2-hydroxyethylsulfonamide* (**IV-4**): white solid, 88.7% yield: m.p. 104–105 °C; IR (KBr) ν_max_ 3215, 3042, 1349, 1320, 1156, 964; ^1^H NMR (400 MHz), δ (ppm) 3.19 (s, 1H, OH), 3.32–3.50 (m, 2H, CH_2_), 5.30–5.32 (q, 1H, CH), 6.92–7.79 (m, 7H, C_6_H_3_ + C_6_H_3_ + NH); Elemental anal. calc. for C_15_H_11_ClF_5_NO_3_S (%): C, 43.33; H, 2.67; N, 3.37; found: C, 43.54; H, 2.39; N, 3.47.

*N-(2-Trifluoromethyl-4-chlorophenyl)-2-(3,5-difluorophenyl)-2-hydroxyethylsulfonamide* (**IV-5**): white solid, 98.8% yield: m.p. 98–100 °C; IR (KBr) ν_max_ 3273, 3025, 1396, 1311, 1122, 989; ^1^H-NMR (400 MHz), δ (ppm) 3.25 (s, 1H, OH), 3.34–3.47(m, 2H, CH_2_), 5.31–5.33 (d, 1H, CH), 6.75–7.78 (m, 7H, C_6_H_3_ + C_6_H_3_ + NH); Elemental anal. calc. for C_15_H_11_ClF_5_NO_3_S (%): C, 43.33; H, 2.67; N, 3.37; found: C, 43.16; H, 2.88; N, 3.24.

#### 4.2.3. General Synthetic Procedure for the Target Compounds **V**

To the solution of *N*-(2-trifluoromethyl-4-chlorophenyl)-2-(3,5-difluorophenyl)-2-hydroxyethyl-sulfonamide (0.01 mol), *N*,*N*,*N′*,*N′*-tetramethylenediamine (TMEDA, 0.006 mol) and 3Å molecular sieves (2 g) in dry CH_2_Cl_2_ (30 mL), acyl chloride (0.011 mol) was added dropwise at room temperature. The mixture was stirred at room temperature for 2 h. The reaction was quenched with ice water (20 mL × 2), filtered and dried over anhydrous magnesium sulfate. After filtering and evaporating the solvent under vacuum, the crude product was purified by silica gel chromatography using petroleum ether/ethyl acetate (10/1, *v*/*v*) as eluent to obtain pure compounds **V-1** to **V-16**.

*N-(2-Trifluoromethyl-4-chlorophenyl)-2-(3,5-difluorophenyl)-2-acetoxyethylsulfonamide* (**V-1**): white solid, 65.5% yield: m.p. 96–98 °C; IR (KBr) ν_max_ 3350, 3097, 2954, 1761, 1307, 1220 cm^−1^; ^1^H-NMR (400 MHz), δ (ppm) 2.11 (s, 3H, CH_3_), 3.38–3.68 (m, 2H, CH_2_), 6.27–6.29 (q, 1H, CH), 7.55–7.58 (d, 1H, NH), 6.78–7.75 (m, 6H, C_6_H_3_ + C_6_H_3_); Elemental anal. calc. for C_17_H_13_ClF_5_NO_4_S (%): C, 44.60; H, 2.86; N, 3.06; found: C, 44.42; H, 3.01; N, 3.25.

*N-(2-Trifluoromethyl-4-chlorophenyl)-2-(3,5-difluorophenyl)-2-benzoyloxyethylsulfonamide* (**V-2**): white solid, 51.8% yield: m.p. 93–95 °C; IR (KBr) ν_max_ 3307, 3032, 2952, 1724, 1338, 1269 cm^−1^; ^1^H-NMR (400 MHz), δ (ppm) 3.53–3.88 (m, 2H, CH_2_), 6.77–6.95 (q, 1H, CH), 6.77–6.95 (q, 3H, CH_3_), 7.55–7.76 (d, 1H, NH), 7.44–7.4 (t, 2H, CH_2_), 7.50–8.01 (m, 6H, C_6_H_3_ + C_6_H_3_); Elemental anal. calc. for C_22_H_15_ClF_5_NO_4_S (%): C, 50.83; H, 2.91; N, 2.69; found: C, 50.67; H, 3.12; N, 2.48.

*N-(2-Trifluoromethyl-4-chlorophenyl)-2-(3,5-difluorophenyl)-2-(2-methylbenzoyloxy)ethylsulfonamide* (**V-3**): white solid, 56.3% yield: m.p. 69–71 °C; IR (KBr) ν_max_ 3325, 3028, 2931, 1747, 1311, 736 cm^−1^; ^1^H-NMR (400 MHz), δ (ppm) 2.54 (s, 3H, CH_3_), 3.51–3.82 (m, 2H, CH_2_), 6.44–6.45 (q, 1H, CH), 6.78–8.05 (m, 11H, C_6_H_3_ + C_6_H_3_ + NH + C_6_H_4_); Elemental anal. calc. for C_23_H_17_ClF_5_NO_4_S (%): C, 51.74; H, 3.21; N, 2.62; found: C, 51.92; H, 3.10; N, 2.81.

*N-(2-Trifluoromethyl-4-chlorophenyl)-2-(3,5-difluorophenyl)-2-(3-methylbenzoyloxy)ethylsulfonamide* (**V-4**): white solid, 81.1% yield: m.p. 114–116 °C; IR (KBr) ν_max_ 3253, 3033, 2945, 1747, 1338, 734 cm^−1^; ^1^H-NMR (400 MHz), δ (ppm) 2.41 (s, 3H, CH_3_), 3.53–3.89 (m, 2H, CH_2_), 6.45–6.48 (q, 1H, CH), 6.77–6.79 (m, 11H, C_6_H_3_ + C_6_H_3_ + NH + C_6_H_4_); Elemental anal. calc. for C_23_H_17_ClF_5_NO_4_S (%): C, 51.74; H, 3.21; N, 2.62; found: C, 51.59; H, 3.43; N, 2.75.

*N-(2-Trifluoromethyl-4-chlorophenyl)-2-(3,5-difluorophenyl)-2-(4-methylbenzoyloxy)ethylsulfonamide* (**V-5**): white solid, 81.8% yield: m.p. 137–139 °C; IR (KBr) ν_max_ 3307, 3032, 2928, 1716, 1338, 827 cm^−1^; ^1^H-NMR (400 MHz), δ (ppm) 2.43 (s, 3H, CH_3_), 3.52–3.87 (q, 2H, CH_2_), 6.45–6.47 (q, 1H, CH), 6.77–7.88 (m, 11H, C_6_H_3_ + C_6_H_3_ + NH + C_6_H_4_); Elemental anal. calc. for C_23_H_17_ClF_5_NO_4_S (%): C, 51.74; H, 3.21; N, 2.62; found: C, 51.88; H, 3.10; N, 2.57.

*N-(2-Trifluoromethyl-4-chlorophenyl)-2-(3,5-difluorophenyl)-2-(4-methoxybenzoyloxy)ethylsulfonamide* (**V-6**): white solid, 60.5% yield: m.p. 118–120 °C; IR (KBr) ν_max_ 3307, 3057, 2943, 1716, 1305, 829 cm^−1^; ^1^H-NMR (400 MHz), δ (ppm) 3.52–3.55 (q, 1H, CH), 3.83–3.87 (q, 1H, CH), 3.88 (s, 3H, CH_3_), 6.43–6.45 (q, 1H, CH), 6.75–7.95 (m, 11H, C_6_H_3_ + C_6_H_3_ + NH + C_6_H_4_); Elemental anal. calc. for C_23_H_17_ClF_5_NO_5_S (%): C, 50.24; H, 3.12; N, 2.55; found: C, 50.49; H, 2.94; N, 2.72.

*N-(2-Trifluoromethyl-4-chlorophenyl)-2-(3,5-difluorophenyl)-2-(2,4-dimethylbenzoyloxy)ethylsulfonamide* (**V-7**): white solid, 71.8% yield: m.p. 120–122 °C; IR (KBr) ν_max_ 3307, 3054, 2927, 1745, 1392, 821 cm^−1^; ^1^H-NMR (400 MHz), δ (ppm) 2.37 (s, 3H, CH3), 2.51 (s, 3H, CH_3_), 3.51–3.85 (m, 2H, CH_2_), 6.42–6.44 (q, 1H, CH), 6.77–7.83 (m, 10H, C_6_H_3_+C_6_H_3_+NH+C_6_H_3_); Elemental anal. calc. for C_24_H_19_ClF_5_NO_4_S (%): C, 52.61; H, 3.50; N, 2.56; found: C, 52.74; H, 3.41; N, 2.68.

*N-(2-Trifluoromethyl-4-chlorophenyl)-2-(3,5-difluorophenyl)-2-(2-chloropropionyloxy)ethylsulfonamide* (**V-8**): light yellow solid, 62.7% yield: m.p. 124–126 °C; IR (KBr) ν_max_ 3502, 3115, 2997, 1691, 1303, 1155 cm^−1^; ^1^H-NMR (400 MHz), δ (ppm) 3.30–3.40 (d, 2H, CH_2_), 3.77–5.82 (m, 6H, CH_2_ + CH_2_ + CH_2_), 6.58–7.85 (m, 7H, C_6_H_3_ + C_6_H_3_ + NH); Elemental anal. calc. for C_18_H_14_Cl_2_F_5_NO_4_S (%): C, 42.70; H, 2.79; N, 2.77; found: C, 42.56; H, 2.91; N, 2.58.

*N-(2-Trifluoromethyl-4-chlorophenyl)-2-(3,5-difluorophenyl)-2-dichloroacetoxyethylsulfonamide* (**V-9**): white solid, 51.4% yield: m.p. 150–152 °C; IR (KBr) ν_max_ 3102, 3010, 2956, 1714, 1384, 1170 cm^−1^; ^1^H-NMR (400 MHz), δ (ppm) 3.93–4.62 (m, 2H, CH_2_), 6.53 (d, 1H, CH), 5.99–6.02 (d, 1H, CH), 6.35–7.88 (m, 7H, C_6_H_3_ + C_6_H_3_ + NH); Elemental anal. calc. for C_17_H_11_Cl_3_F_5_NO_4_S (%): C, 38.77; H, 2.11; N, 2.66; found: C, 38.86; H, 2.33; N, 2.48.

*N-(2-Trifluoromethyl-4-chlorophenyl)-2-(3,5-difluorophenyl)-2-trichloroacetoxyethylsulfonamide* (**V-10**): white solid, 80.9% yield: m.p. 168–170 °C; IR (KBr) ν_max_ 3082, 3010, 2954, 1705, 1379, 1224 cm^−1^; ^1^H-NMR (400 MHz), δ (ppm) 4.05–4.71 (m, 2H, CH_2_), 6.32–6.34 (q, 1H, CH), 6.88–7.79 (m, 7H, C_6_H_3_ + C_6_H_3_ + NH); Elemental anal. calc. for C_17_H_10_Cl_4_F_5_NO_4_S (%): C, 36.39; H, 1.80; N, 2.50; found: C, 36.51; H, 2.02; N, 2.31.

*N-(2-Trifluoromethyl-4-chlorophenyl)-2-(3,5-difluorophenyl)-2-(2-fluorobenzoyloxy)ethylsulfonamide* (**V-11**): white solid, 75.6% yield: m.p. 118–120 °C; IR (KBr) ν_max_ 3307, 3095, 2956, 1743, 756, 686 cm^−1^; ^1^H-NMR (400 MHz), δ (ppm) 3.52–3.88 (m, 2H, CH_2_), 6.49–6.51 (q, 1H, CH), 6.78–7.93 (m, 11H, C_6_H_3_ + C_6_H_3_ + NH + C_6_H_4_); Elemental anal. calc. for C_22_H_14_ClF_6_NO_4_S (%): C, 49.13; H, 2.62; N, 2.60; found: C, 49.32; H, 2.51; N, 2.84.

*N-(2-Trifluoromethyl-4-chlorophenyl)-2-(3,5-difluorophenyl)-2-(3-fluorobenzoyloxy)ethylsulfonamide* (**V-12**): white solid, 32.9% yield: m.p. 97–99 °C; IR (KBr) ν_max_ 3290, 3099, 2953, 1741, 752, 684 cm^−1^; ^1^H-NMR (400 MHz), δ (ppm) 3.51–3.97 (m, 2H, CH_2_), 6.45–6.47 (q, 1H, CH), 6.77–7.81 (m, 11H, C_6_H_3_ + C_6_H_3_ + NH + C_6_H_4_); Elemental anal. calc. for C_22_H_14_ClF_6_NO_4_S (%): C, 49.13; H, 2.62; N, 2.60; found: C, 48.97; H, 2.54; N, 2.77.

*N-(2-Trifluoromethyl-4-chlorophenyl)-2-(3,5-difluorophenyl)-2-(2-chlorobenzoyloxy)ethylsulfonamide* (**V-13**): white solid, 49.6% yield: m.p. 99–101 °C; IR (KBr) ν_max_ 3327, 3080, 2931, 1741, 754, 692 cm^−1^; ^1^H-NMR (400 MHz), δ (ppm) 3.52–3.87 (m, 2H, CH_2_), 6.45–6.48 (q, 1H, CH), 6.87–7.90 (m, 11H, C_6_H_3_ + C_6_H_3_ + NH + C_6_H_4_); Elemental anal. calc. for C_22_H_14_Cl_2_F_5_NO_4_S (%): C, 47.67; H, 2.55; N, 2.53; found: C, 47.43; H, 2.38; N, 2.69.

*N-(2-Trifluoromethyl-4-chlorophenyl)-2-(3,5-difluorophenyl)-2-(3-chlorobenzoyloxy)ethylsulfonamide* (**V-14**): white solid, 69.5% yield: m.p. 114–116 °C; IR (KBr) ν_max_ 3273, 3048, 2935, 1747, 1338, 742 cm^−1^; ^1^H-NMR (400 MHz), δ (ppm) 3.52–3.88 (m, 2H, CH_2_), 6.45–6.48 (q, 1H, CH), 6.78–7.95 (m, 11H, C_6_H_3_ + C_6_H_3_ + NH + C_6_H_4_); Elemental anal. calc. for C_22_H_14_Cl_2_F_5_NO_4_S (%): C, 47.67; H, 2.55; N, 2.53; found: C, 47.45; H, 2.78; N, 2.39.

*N-(2-Trifluoromethyl-4-chlorophenyl)-2-(3,5-difluorophenyl)-2-(2-trifluoromethylbenzoyloxy) ethylsulfonamide* (**V-15**): white solid, 79.8% yield: m.p. 81–83 °C; IR (KBr) ν_max_ 3308, 3095, 2948, 1747, 1319, 779 cm^−1^; ^1^H-NMR (400 MHz), δ (ppm) 3.51–3.84 (m, 2H, CH_2_), 6.45–6.48 (q, 1H, CH), 6.75–7.90 (m, 11H, C_6_H_3_ + C_6_H_3_ + NH + C_6_H_4_); Elemental anal. calc. for C_23_H_14_ClF_8_NO_4_S (%): C, 46.99; H, 2.40; N, 2.38; found: C, 46.78; H, 2.64; N, 2.14.

*N-(2-Trifluoromethyl-4-chlorophenyl)-2-(3,5-difluorophenyl)-2-(3-trifluoromethylbenzoyloxy) ethylsulfonamide* (**V-16**): white solid, 43.9% yield: m.p. 119–121 °C; IR (KBr) ν_max_ 3273, 3054, 2931, 1747, 1338, 694 cm^−1^; ^1^H-NMR (400 MHz), δ (ppm) 3.52–4.15 (m, 2H, CH_2_), 6.49–6.51 (q, 1H, CH), 6.79–8.27 (m, 11H, C_6_H_3_ + C_6_H_3_ + NH + C_6_H_4_); Elemental anal. calc. for C_23_H_14_ClF_8_NO_4_S (%): C, 46.99; H, 2.40; N, 2.38; found: C, 47.11; H, 2.26; N, 2.51.

### 4.3. Fungicidal Activity Bioassays

The in vitro and in vivo fungicidal activities of all the title compounds against *B. cinerea* were tested by a mycelium growth inhibition assay and greenhouse pot experiments, respectively. The *Botrytis cinerea* strains (**DL-11**, **HLD-15**) were isolated from damaged parts of tomato in a greenhouse in different areas of Liaoning, China, and cultured on potato dextrose agar (PDA) for many generations. The commercial fungicides procymidone (with a purity of 96%), pyrimethanil (with a purity of 95%) and chlorothalonil (with a purity of 96%) provided by the Shenyang Research Institute of the Chemical Industry, National Pesticides Engineering Research Centre, were used as positive controls. Excel 2007 (Microsoft, Redmond, WA, USA) was used to analyze the bioassay data. Analysis of difference significances was performed using SPSS v.18.0 (SPSS Inc., Chicago, IL, USA).

#### 4.3.1. Evaluation of Compounds **III**, **IV**, **V** on the Mycelia Growth of *Botrytis cinerea* in Solid Media

The in vitro fungicidal activity of compounds **III**, **IV** and **V** was assessed using a radial growth test on potato dextrose agar (PDA). The compounds were dissolved in acetone and mixed with sterile molten PDA to obtain five concentrations of 100, 25, 6.25, 1.56 and 0.39 mg L^−1^. PDA with different concentrations of the test compounds was poured into 90 mm Petri dishes (15 mL per dish), which were then inoculated with 5 mm plugs of *B. cinerea*. The plugs were obtained from a PDA culture plate by punching at the edge of the mycelia colony. Three replicates were used per treatment. The commercial fungicides were used as positive controls. After an incubation period of 72 h at 23 °C under a regular 12:12 h light:dark regimen, mycelia growth diameters were measured and the inhibition percentages relative to the control with 1% acetone were calculated. The inhibition rate was determined following a standard method [35]. The EC_50_ values were calculated using log-probit analysis.

#### 4.3.2. In Vivo Fungicidal Activity against *Botrytis cinerea* by Greenhouse Pot Experiments

The in vivo fungicidal activity of the title compounds against *B. cinerea* was evaluated in the greenhouse. *B. cinerea* was maintained on PDA medium at 4 °C. The culture plates were cultivated at 24 ± 1 °C. Germination was conducted by soaking cucumber seeds in water for 2 h at 50 °C and then keeping the seeds moist for 24 h at 28 °C in an incubator. When the radicles were 0.5 cm, the seeds were grown in plastic pots containing a 1:1 (*v*/*v*) mixture of vermiculite and peat. Cucumber plants used for inoculations were at the stage of two seed leaves. The tested compounds were confected to 2.5% EC formulations, which were diluted to concentration of 500 μg/mL with water to obtain the solutions. Tested compounds and commercial fungicides were sprayed with a hand spray on the surface of the seed leaves. Water sprayed seed leaves were set as the CK. After drying, the upper sides of the leaves were inoculated with 5 mm plugs of *B. cinerea*, which was maintained on PDA. The plants were maintained at 24 ± 1 °C and above 80% relative humidity in the greenhouse. The fungicidal activity was then evaluated.

#### 4.3.3. In vitro Fungicidal Activity of Compounds **III**, **IV**, **V** against Different Phytopathogenic Fungi

The fungicidal activity of compounds **III**, **IV** and **V** against phytopathogentic fungi (*Pyricularia grisea*, *Exserohilum turcicum* (Pass.) Leonard et Suggs, *Pythium aphanidermatum*, *Phytophthora capsici* Leonian, *Fusarium graminearum* Schw., *Corynespora cassiicola* and *Thanatephorus cucumeris*) was assessed using the mycelium growth test on PDA. The compounds were dissolved in acetone and mixed PDA to obtain final concentration of 50 mg L^−1^. Chlorothalonil was used as the positive control. Other test conditions were the same with the method given in the mycelial growth.

## 5. Conclusions

In summary, 38 2-substituted-phenyl-2-oxo-, 2-hydroxy- and 2-acyloxyethylsulfonamides were designed and synthesized. Their structures were characterized by ^1^H-NMR, IR and elemental analysis. In vitro and in vivo fungicidal activities against 2 different *B. cinerea* strains were tested. The results showed that some title compounds exhibited better fungicidal activities than that of the lead compound **B-1**. Among them, compound **V-13** showed better in vivo fungicidal activity than the commercial fungicides procymidone and pyrimethanil. In addition, these new compounds had a broad spectrum of fungicidal activity. Bioassays showed that some target molecules might be used as new lead compounds for further development of novel fungicides against *B. cinerea*. Finally, as we all know, sulfonamides are more used as herbicides, while its application as fungicides is less common in agrochemicals. In our preliminary research, we found that *Botrytis cinerea* control using sulfonamides may involve a new mode of action mode [36,37], which makes our research more significant. Hence, the enhancement of the activity of these sulfonamide fungicides will be our focus in further research.

## Figures and Tables

**Table 1:** The EC_50_ values of all the title compounds against 2 kinds of *B. cinerea* in vitro.

Compd.	DL-11		HLD-15	
	EC_50_ (mg L^−1^)	95% Confidence Limit (mg L^−1^)	EC_50_ (mg L^−1^)	95% Confidence Limit (mg L^−1^)
**III-1**	8.67	6.41–11.73	16.25	11.82–22.33
**III-2**	10.81	6.81–17.16	17.70	10.99–28.51
**III-3**	7.51	5.14–10.99	8.12	6.22–10.61
**III-4**	8.54	6.38–11.44	8.61	6.01–12.33
**III-5**	5.44	3.18–9.30	7.94	5.83–10.82
**III-6**	8.60	6.71–11.00	7.91	5.89–10.62
**III-7**	3.98	2.86–5.52	8.00	6.05–10.58
**III-8**	2.68	1.96–3.67	4.16	2.82–6.15
**III-9**	5.15	3.79–7.01	7.22	5.01–10.40
**III-10**	3.82	2.58–5.65	5.40	3.66–7.97
**III-11**	4.07	2.95–5.59	6.83	4.83–9.67
**III-12**	4.69	3.05–7.23	6.89	5.10–9.31
**III-13**	1.58	1.03–2.43	3.48	2.37–5.12
**III-14**	2.02	1.68–2.36	3.97	2.76–5.71
**III-15**	1.64	1.15–2.34	8.53	5.16–14.12
**III-16**	5.67	4.19–7.67	6.48	4.69–8.95
**III-17**	7.15	5.29–9.67	7.74	5.45–10.99
**B-1**	14.25	10.08–19.02	22.79	19.22–27.74
**IV-1**	13.33	10.29–17.27	33.16	23.80–46.20
**IV-2**	3.01	2.25–4.03	7.17	5.50–9.34
**IV-3**	3.50	2.59–4.72	4.07	2.92–5.68
**IV-4**	0.74	0.38–1.44	2.49	1.72–3.59
**IV-5**	0.70	0.38–1.30	0.61	0.28–1.35
**V-1**	0.10	0.02–0.45	3.32	2.26–4.88
**V-2**	1.96	1.18–3.25	4.69	3.05–7.23
**V-3**	6.10	4.01–9.27	132.52	54.23–323.82
**V-4**	14.27	9.43–21.58	3648.50	197.38–67442.80
**V-5**	1383.25	116.31–16450.90	1443.05	184.66–11277.00
**V-6**	131.12	39.92–430.68	/	/
**V-7**	11.72	7.33–18.73	9.80	5.63–17.07
**V-8**	1.32	0.80–2.17	13.80	9.20–20.71
**V-9**	0.01	0.00–0.22	7.72	5.81–10.25
**V-10**	1.18	0.69–2.00	4.73	3.36–6.67
**V-11**	5.82	3.84–8.82	119.32	44.64–318.93
**V-12**	6.99	5.14–9.51	21.09	14.97–29.72
**V-13**	35.18	14.78–83.75	825.50	111.18–6129.14
**V-14**	23.63	17.45–32.00	338.33	130.28–780.13
**V-15**	20.33	10.69–38.66	553.23	144.46–2118.63
**V-16**	1279.16	117.52–13923.30	139.55	71.51–272.32
procymidone	2.59	1.78–3.76	15.95	11.78–21.60
chlorothalonil	1.66	0.97–2.84	17.52	8.04–38.19

**Table 2:** Control efficiency of all the title compounds against *B. cinerea* in vivo (500 mg L^−1^).

Compd.	Control Efficiency (%) against Strain DL-11 ± SEM	Compd.	Control Efficiency (%) against Strain DL-11 ± SEM
**III-1**	54.9 ± 9.1	**IV-3**	58.2 ± 7.3
**III-2**	50.5 ± 9.5	**IV-4**	67.1 ± 8.2
**III-3**	65.9 ± 8.1	**IV-5**	62.9 ± 7.6
**III-4**	60.4 ± 8.6	**V-1**	70.8 ± 15.4
**III-5**	52.7 ± 9.3	**V-2**	69.2 ± 13.9
**III-6**	40.7 ± 10.4	**V-3**	67.1 ± 4.2
**III-7**	25.3 ± 11.8	**V-4**	68.3 ± 9.7
**III-8**	57.1 ± 8.9	**V-5**	61.45 ± 6.0
**III-9**	44.0 ± 10.1	**V-6**	71.3 ± 5.2
**III-10**	47.3 ± 9.8	**V-7**	68.3 ± 9.7
**III-11**	58.2 ± 8.8	**V-8**	70.8 ± 10.4
**III-12**	58.2 ± 8.8	**V-9**	65.4 ± 9.0
**III-13**	62.6 ± 8.4	**V-10**	71.3 ± 14.6
**III-14**	33.0 ± 11.1	**V-11**	66.7 ± 4.8
**III-15**	53.8 ± 9.3	**V-12**	68.3 ± 5.5
**III-16**	57.1 ± 8.9	**V-13**	74.6 ± 11.5
**III-17**	42.9 ± 10.2	**V-14**	73.3 ± 11.8
**IV-1**	67.1 ± 8.7	**V-15**	67.1 ± 9.4
**IV-2**	52.9 ± 7.4	**V-16**	67.9 ± 8.0
procymidone	72.5 ± 6.1	pyrimethanil	61.7 ± 8.1

**Table 3:** The EC_50_ values of selected compounds **III**, **IV** and **V** against different phytopathogenic fungi in vitro.

Compd.	EC_50_ (mg L^−1^)			
	Fg ^a^	Tc ^a^	Pa ^a^	Pc ^a^
**III-1**	8.27	1.39	10.49	3.7
**III-2**	5.12	4.02	9.36	4.17
**III-3**	4.72	1.65	14.53	3.06
**III-4**	3.55	3.07	13.41	2.77
**III-6**	1.41	0.99	7.25	2.25
**III-10**	1.05	0.60	8.65	4.47
**III-12**	2.84	2.46	11.42	6.02
**III-13**	0.79	1.86	8.62	2.74
**III-16**	3.26	3.67	11.17	4.26
**IV-5**	3.17	1.88	6.52	4.44
**V-1**	7.48	15.2	27.33	28.4
**V-8**	4.59	1.52	10.72	4.79
**V-9**	9.8	1.57	19.94	5.6
**V-10**	2.41	1.07	6.96	3.22
**B-1**	4.13	2.33	16.92	3.18
chlorothalonil	0.35	0.02	5.93	17.22

^a^ Fg = *Fusarium graminearum Schw*.; Tc = *Thanatephorus cucumeris*; Pa = *Pythium aphanidermatum*; Pc = *Phytophthora capsici Leonian*.

## Supplementary Materials

Supplementary materials are available online. The ^1^H-NMR spectrogram of compounds III, IV and V.
