# Supplementary material for: Design, Synthesis and Fungicidal Activity of 2-Substituted Phenyl-2-oxo-, 2-Hydroxy- and 2-Acyloxyethylsulfonamides
Source: Molecules. 2017 May 4;22(5):738. doi: 10.3390/molecules22050738 (PMC6154733; doi:10.3390/molecules22050738)
Supplement: Supplementary file 1 [file molecules-22-00738-s001.pdf]

Design, Synthesis and Fungicidal Activity of 2-Substituted Phenyl-2-oxo-, 2-Hydroxy- and 2-Acyloxyethylsulfonamides

Minlong Wang [1,†], Peng Rui [1,†], Caixiu Liu [1], Ying Du [1], Peiwen Qin [1], Zhiqiu Qi [1], Mingshan Ji [1], Xinghai Li [1,*] and Zining Cui [2,3,*]

[1] Department of Pesticide Science, Plant Protection College, Shenyang Agricultural University, Shenyang 110866, China; wangminlong0906@163.com (M.W.); ruiace@126.com (P.R.); LCX14714027@163.com (C.L.); duying92m@163.com (Y.D.); qinpeiwen08@sina.com (P.Q.); syqizhiqiu@sina.com (Z.Q.); jimingshan@163.com (M.J.)

[2] State Key Laboratory for Conservation and Utilization of Subtropical Agro-Bioresources, Integrative Microbiology Research Centre, Guangdong Province Key Laboratory of Microbial Signals and Disease Control, South China Agricultural University, Guangzhou 510642, China

[3] Key Laboratory of Green Pesticide and Agricultural Bioengineering, Ministry of Education, Guizhou University, Guiyang 550025, China

* Correspondence: xinghai30@163.com (X.L.); ziningcui@scau.edu.cn (Z.C.); Tel.: +86-24-8834-2018 (X.L); +86-20-8528-8229 (Z.C.)

† These authors contributed equally to this work.

Pages S2-S10: ^{1}H-NMR spectra of title compounds **III1-III17**

Pages S11-S13: ^{1}H-NMR spectra of title compounds **IV1-IV5**

Pages S14-S21: ^{1}H-NMR spectra of title compounds **V1-V16**

^{1}H-NMR spectra of title compounds **III1-III17**

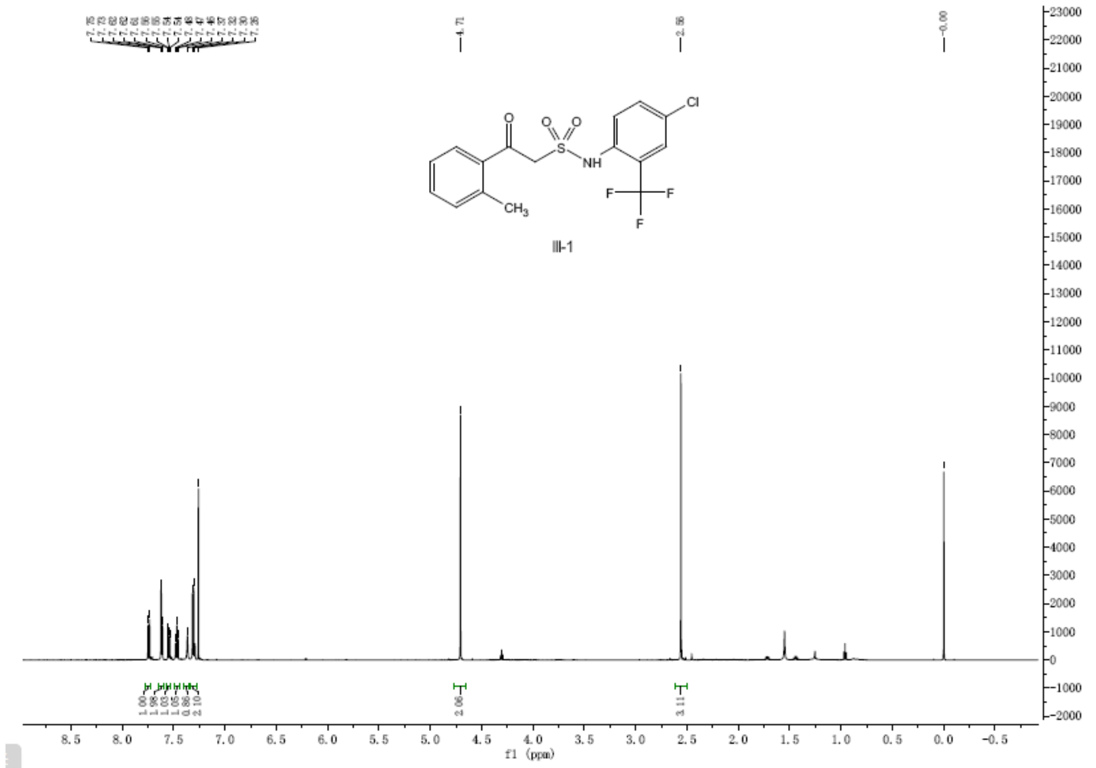

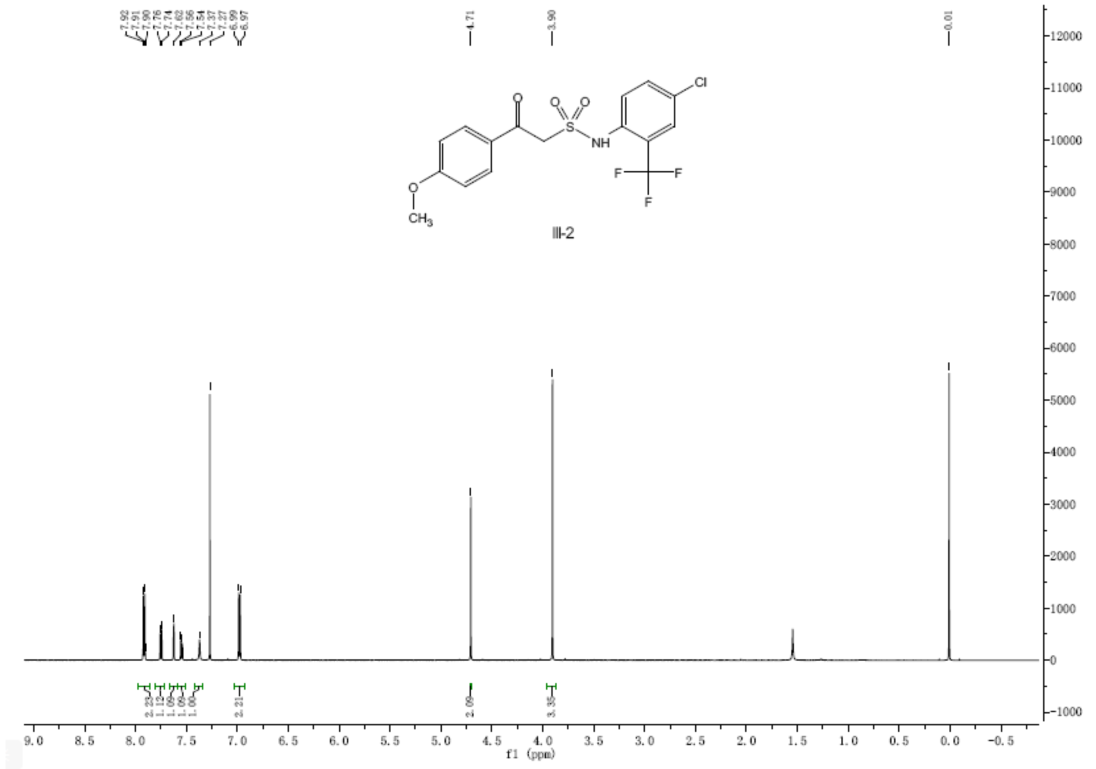

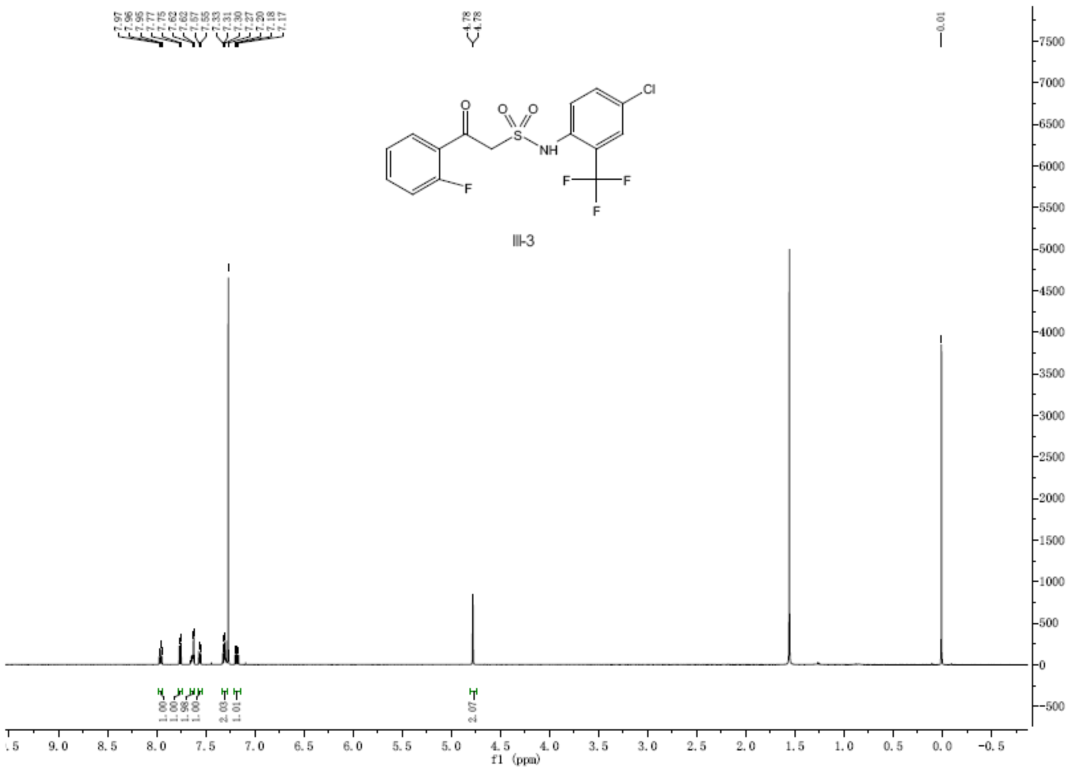
Cl
NH
F
III-3
f1 (ppm)

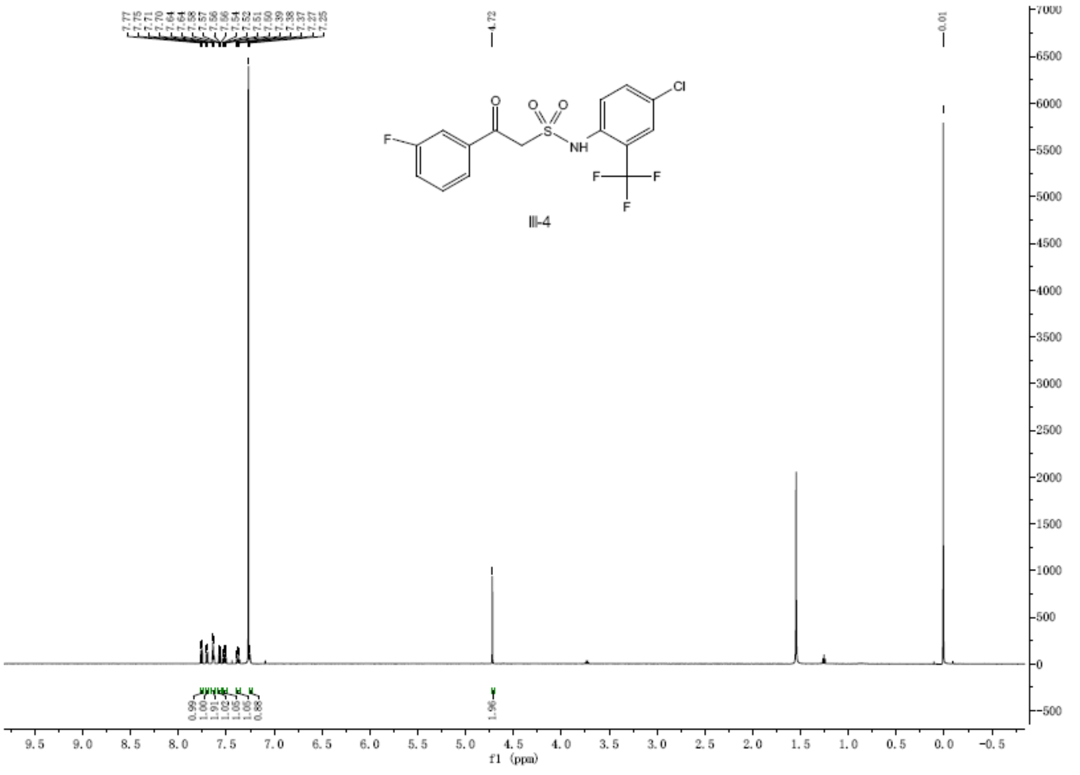
Cl
NH
F
III-4
f1 (ppm)

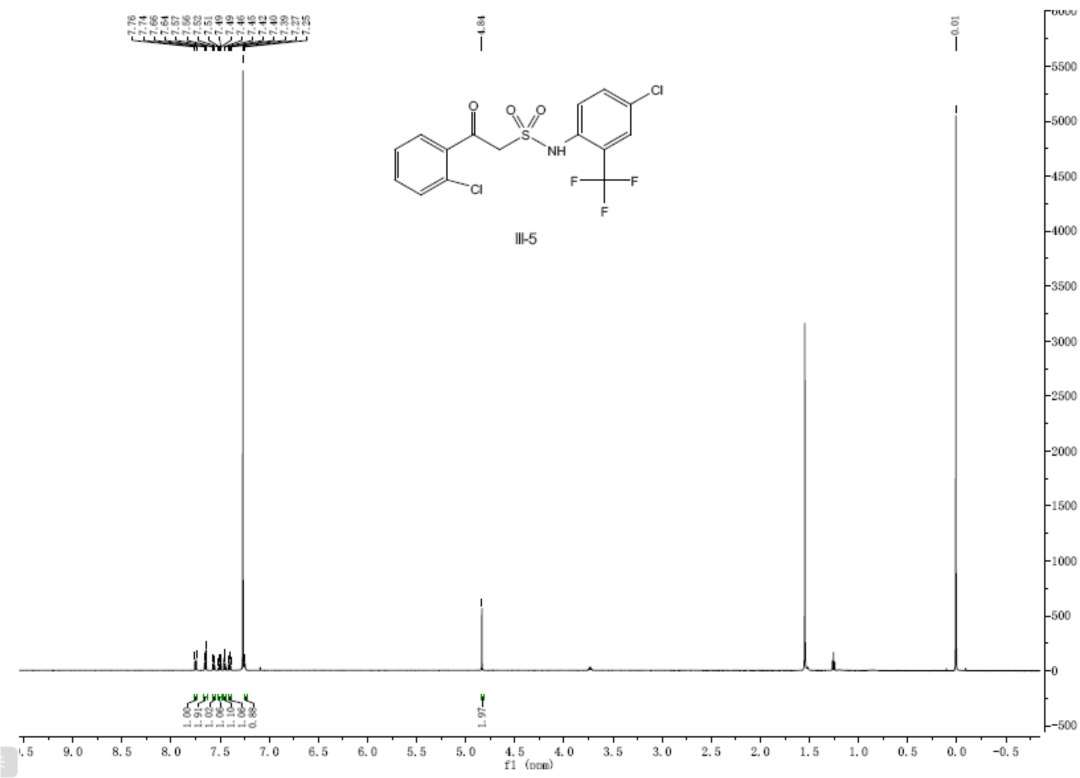
III-5
f1 (ppm)

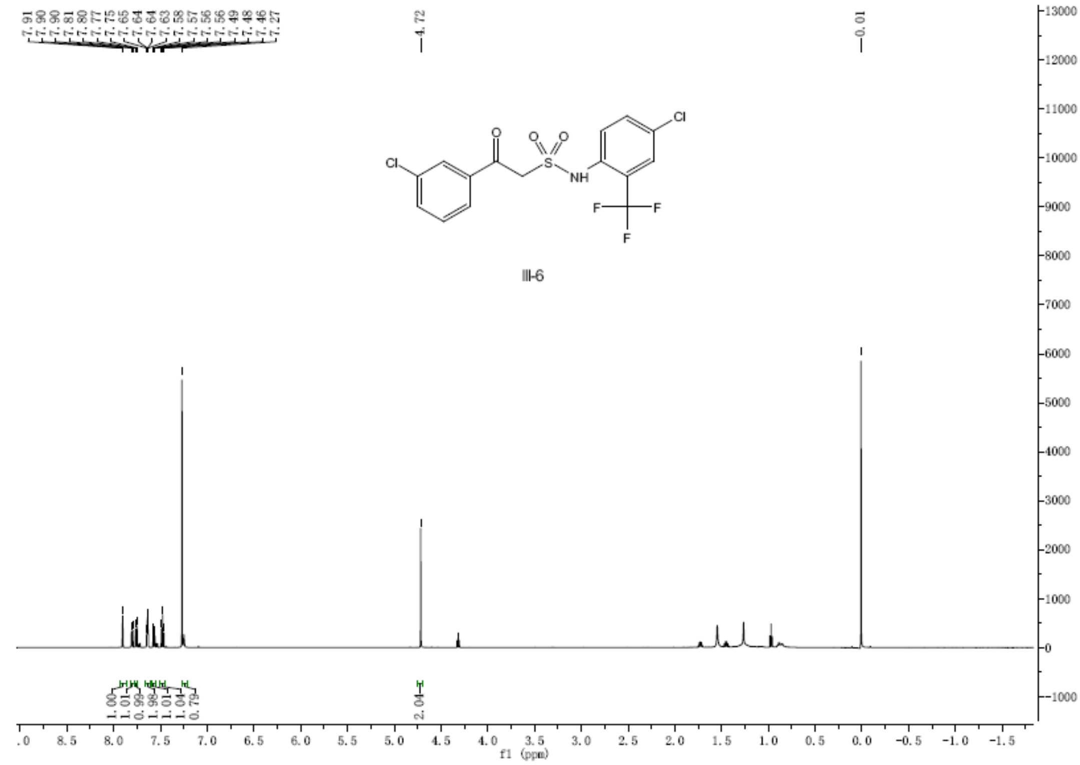
III-6
f1 (ppm)

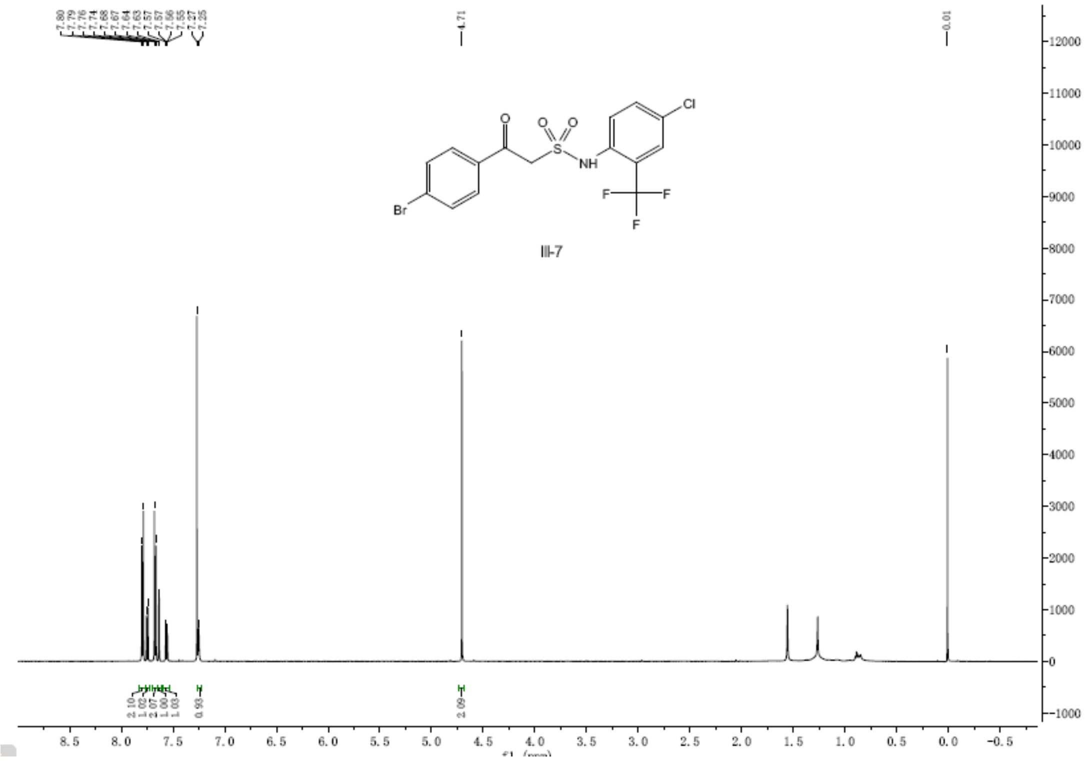
III-7
f1 (ppm)

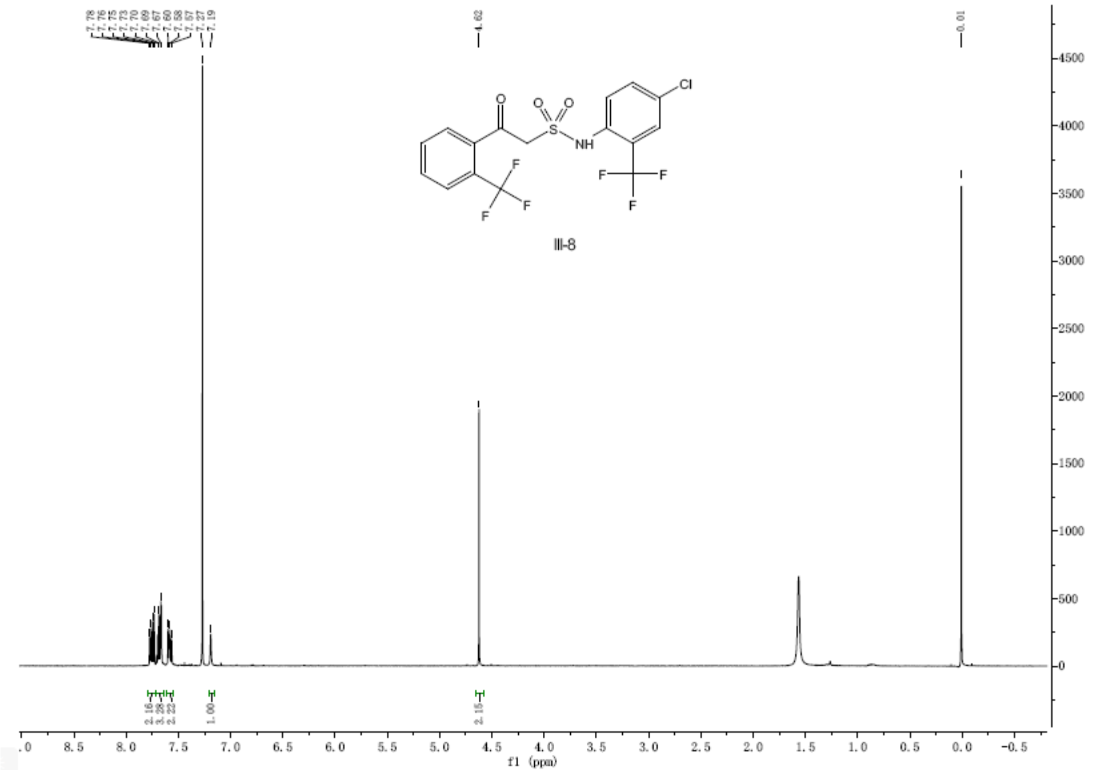
III-8
f1 (ppm)

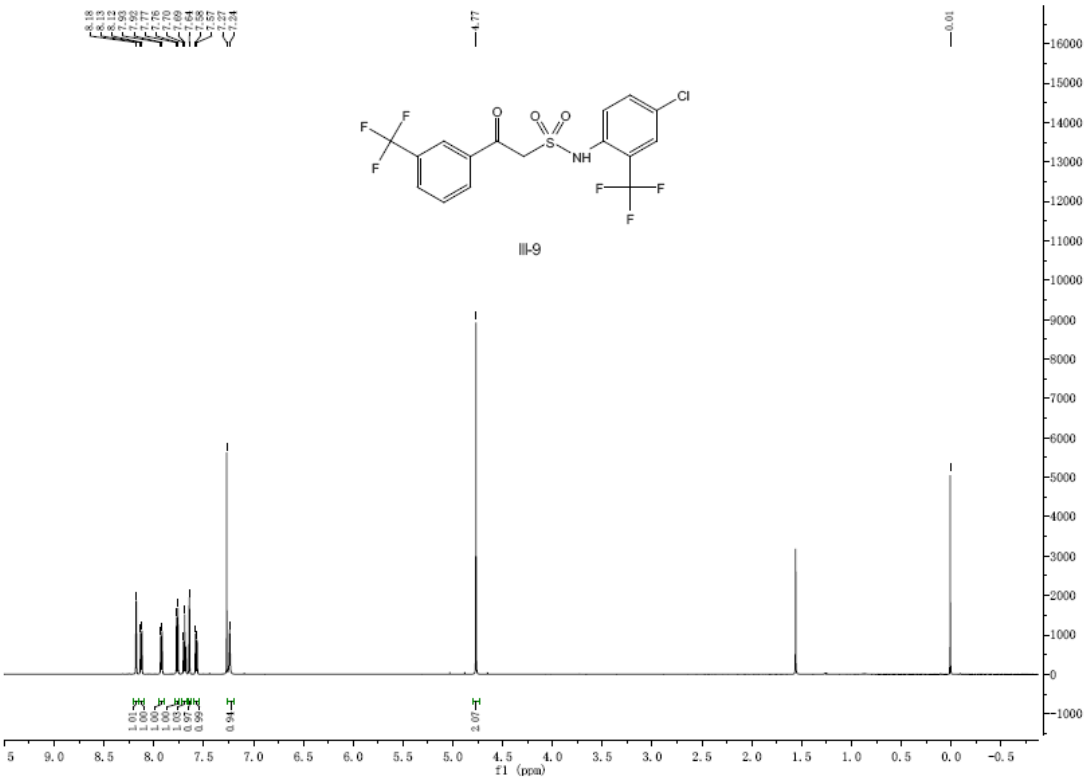
III-9
f1 (ppm)

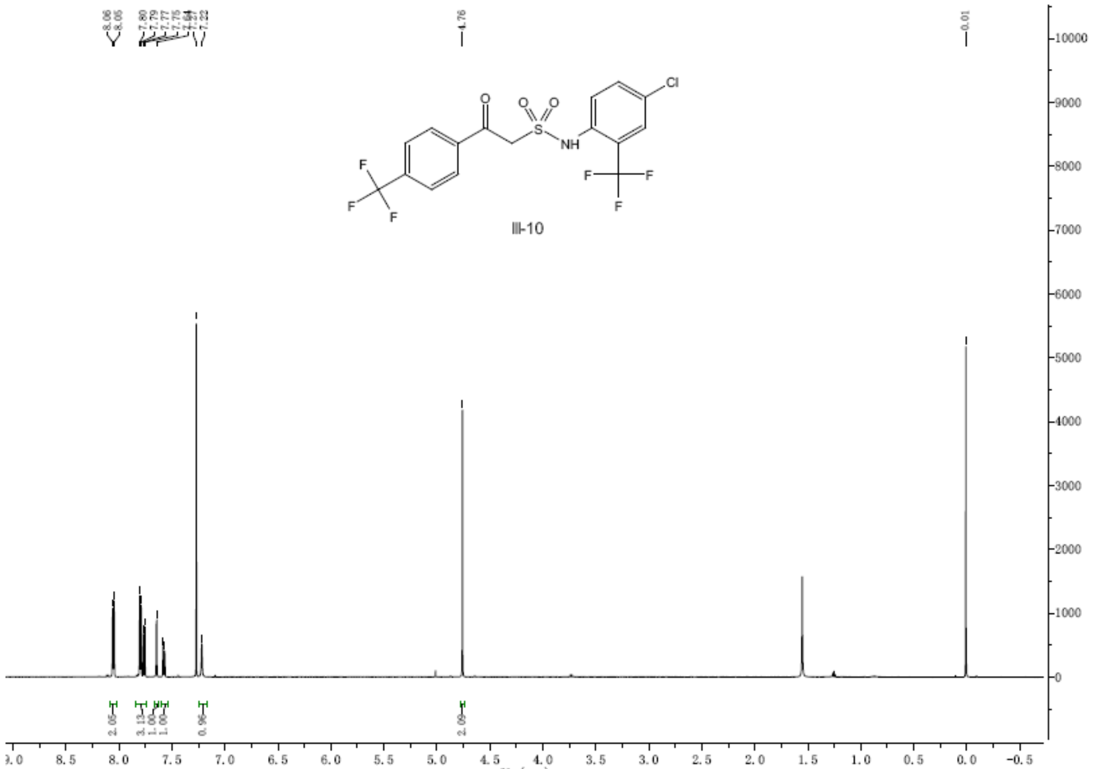
III-10
f1 (ppm)

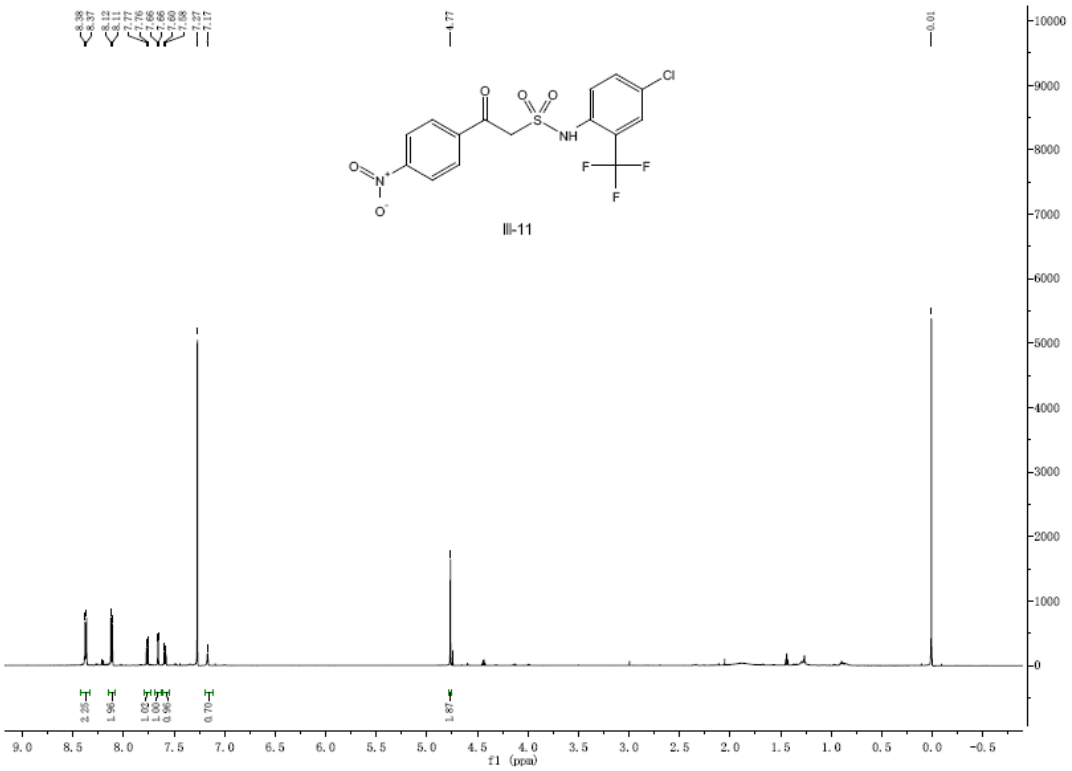
III-11
f1 (ppm)

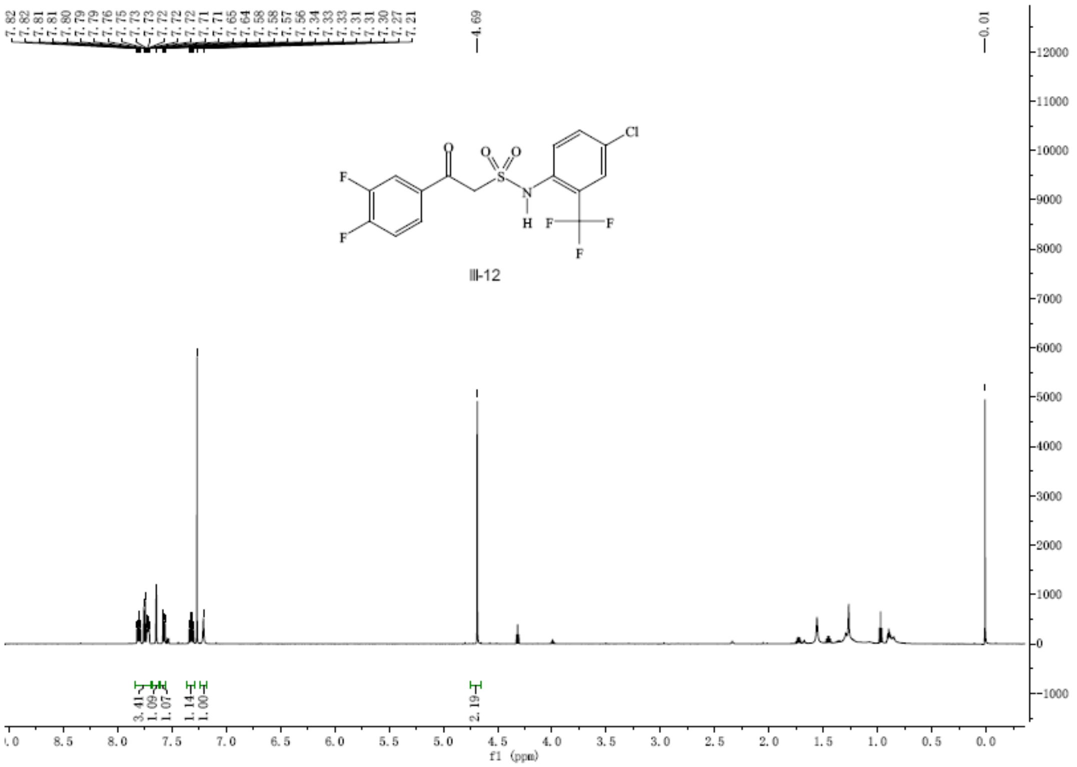
III-12
f1 (ppm)

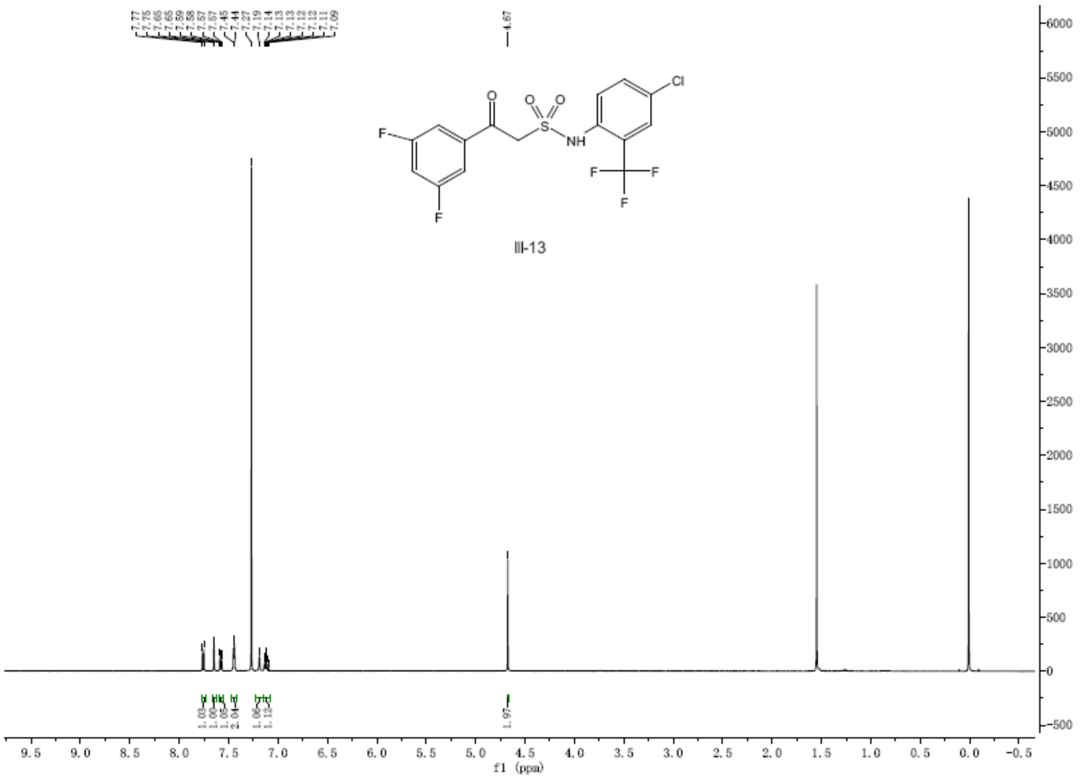
III-13
f1 (ppm)

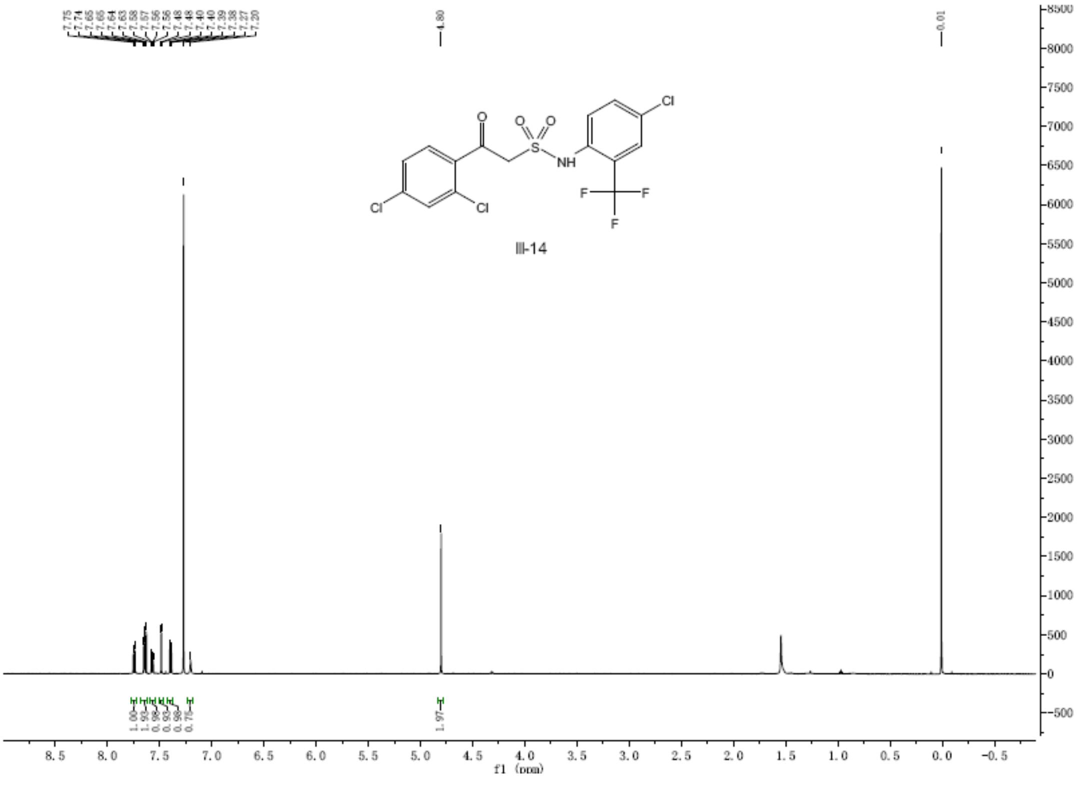
III-14
f1 (ppm)

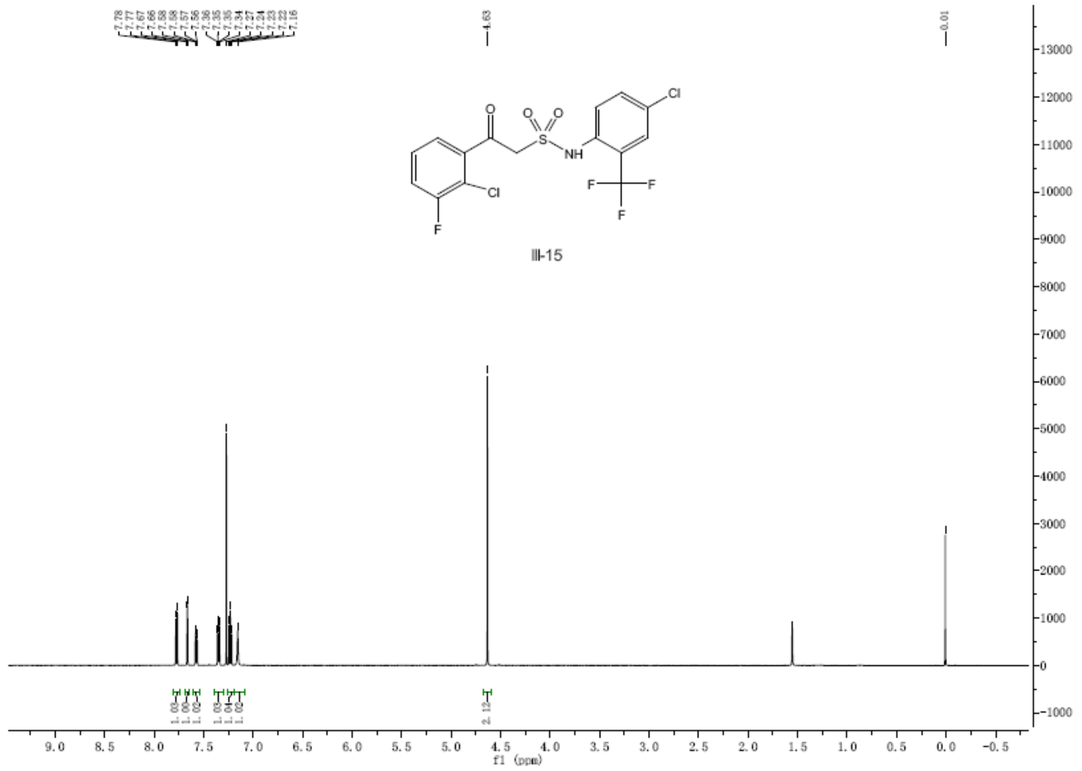
III-15
4.63
0.01
f1 (ppm)

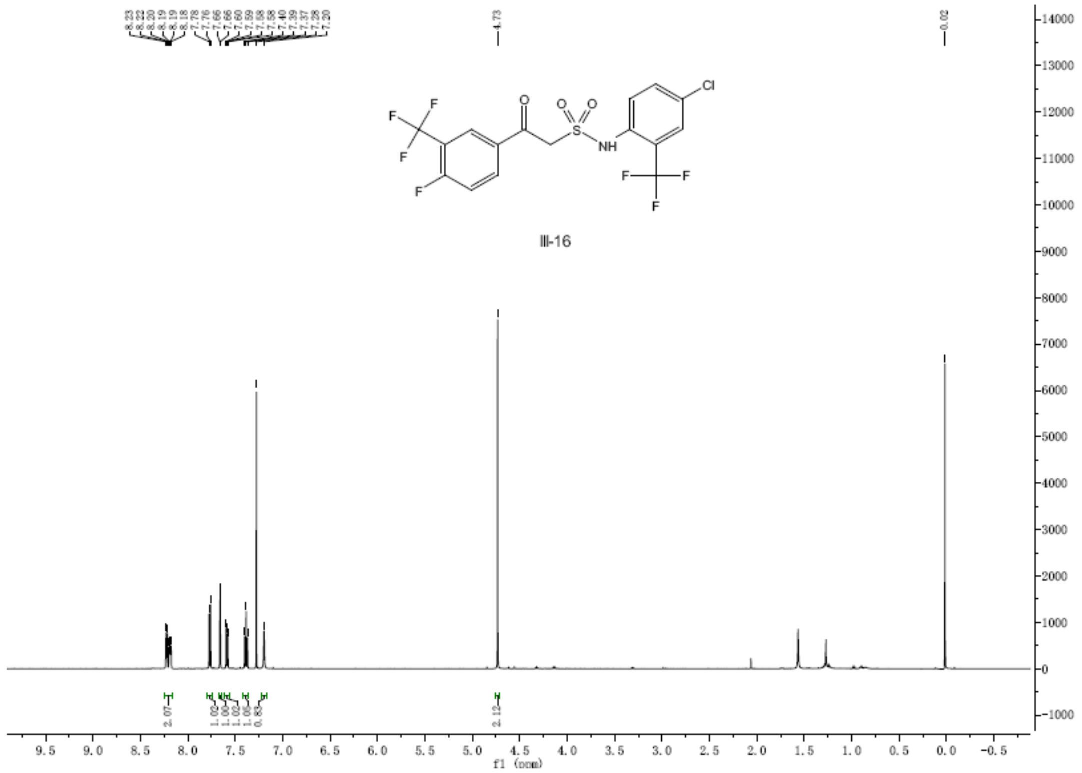
III-16
4.73
0.02
f1 (ppm)

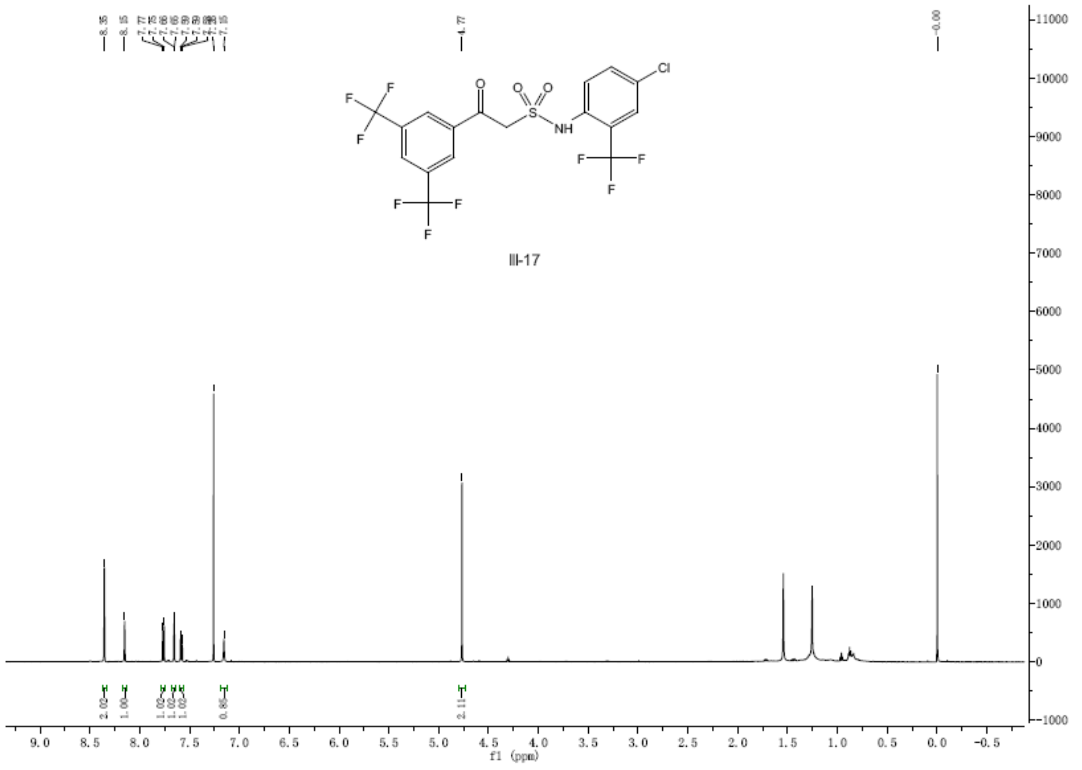

^{1}H-NMR spectra of title compounds **IV1-IV5**

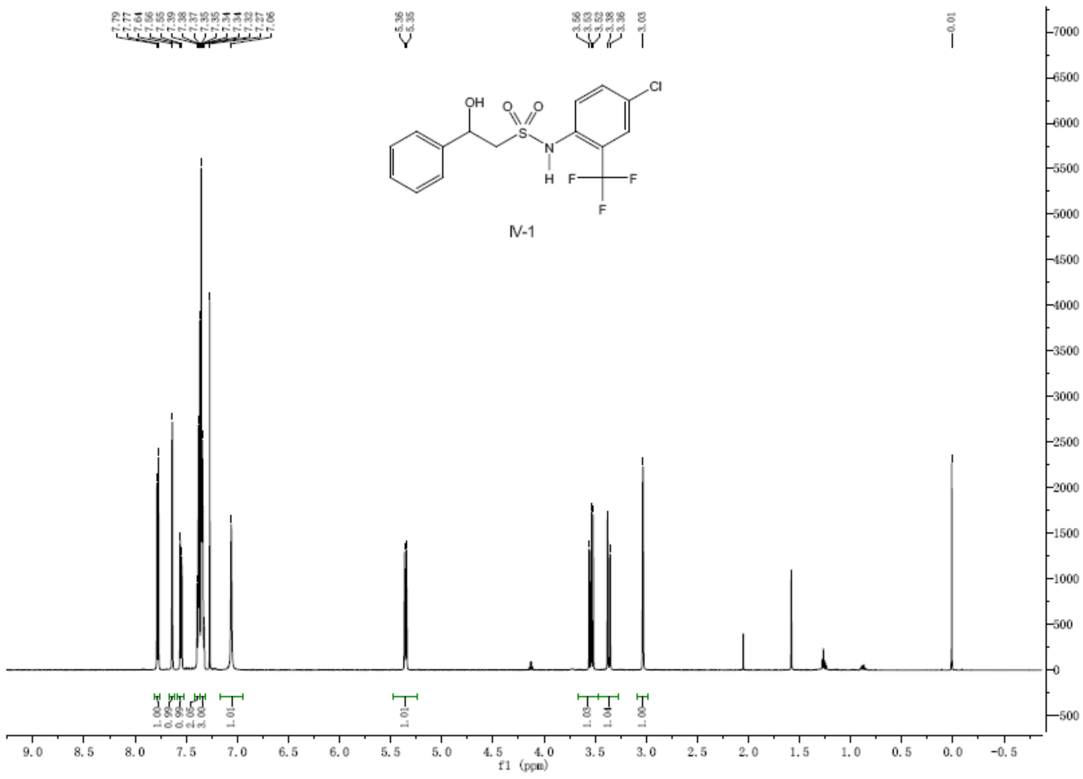

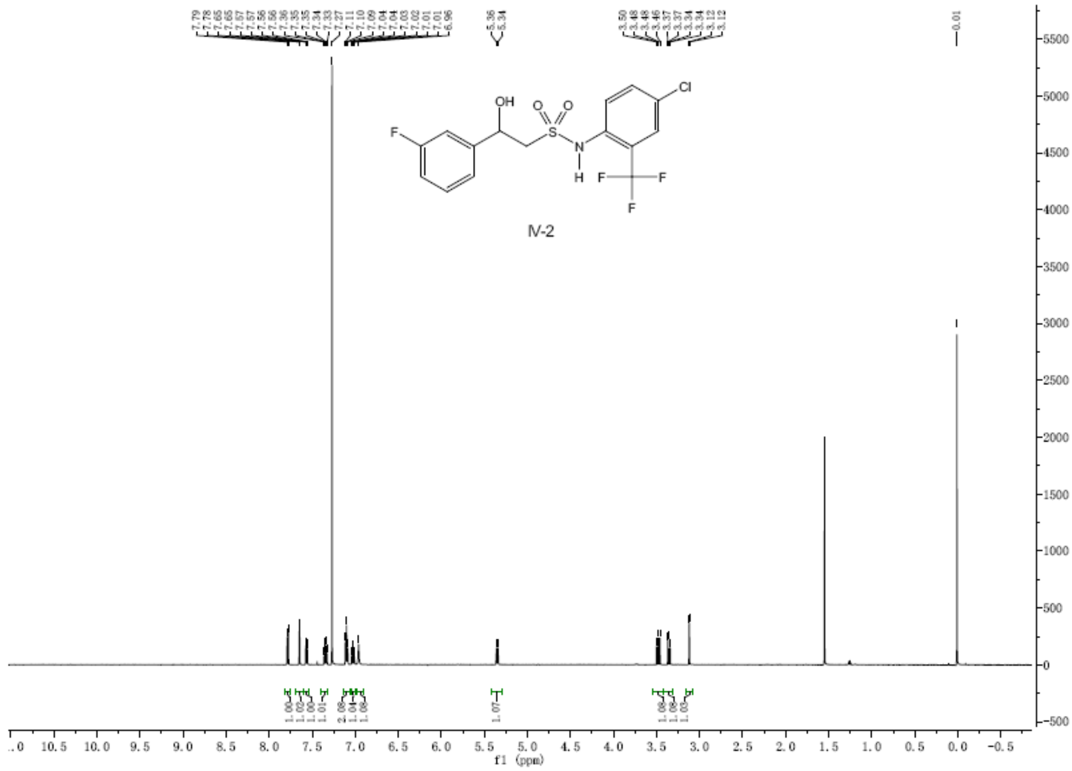

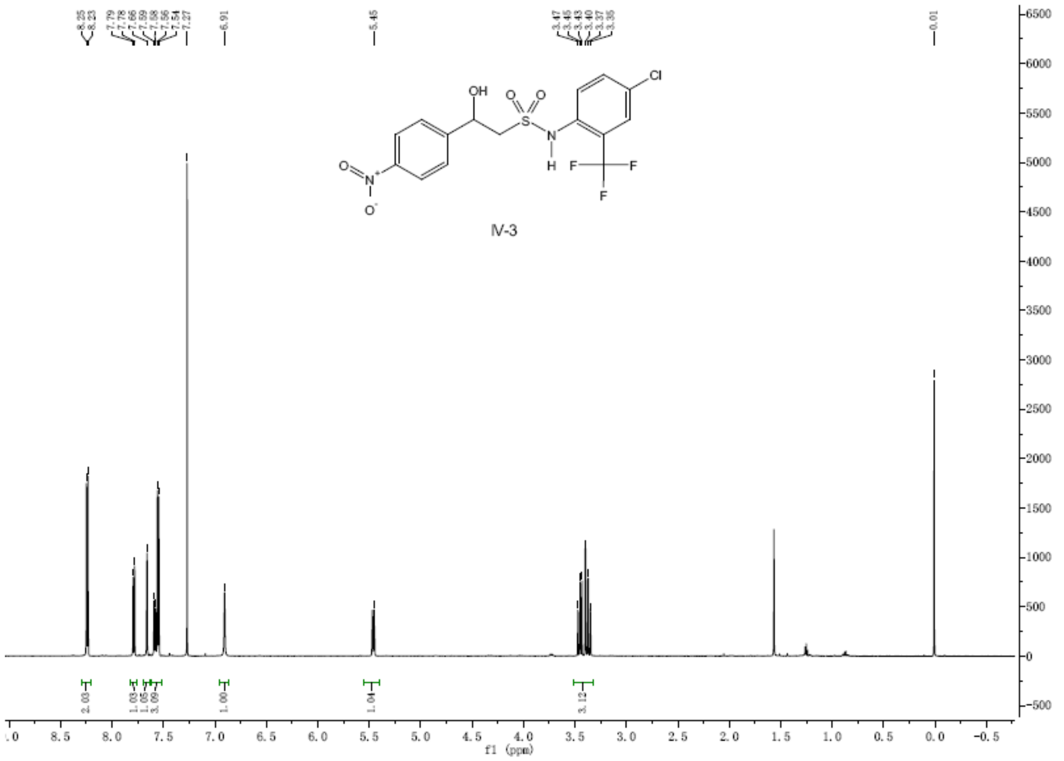
IV-3
f1 (ppm)

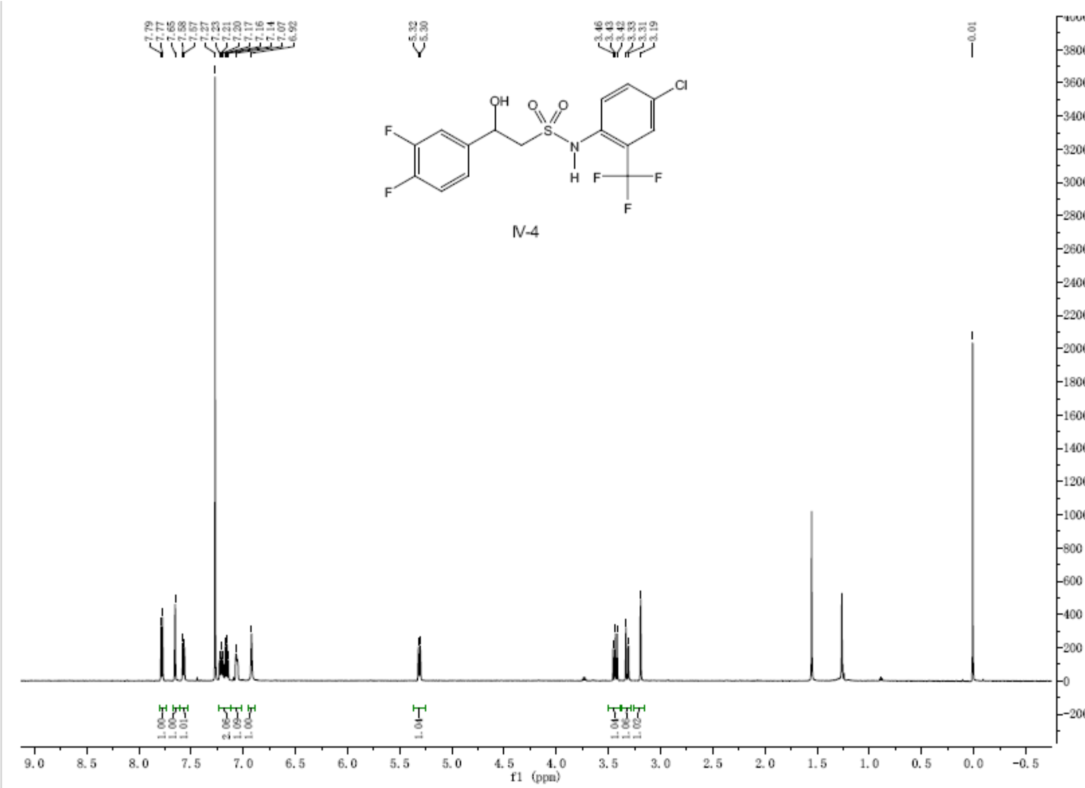
IV-4
f1 (ppm)

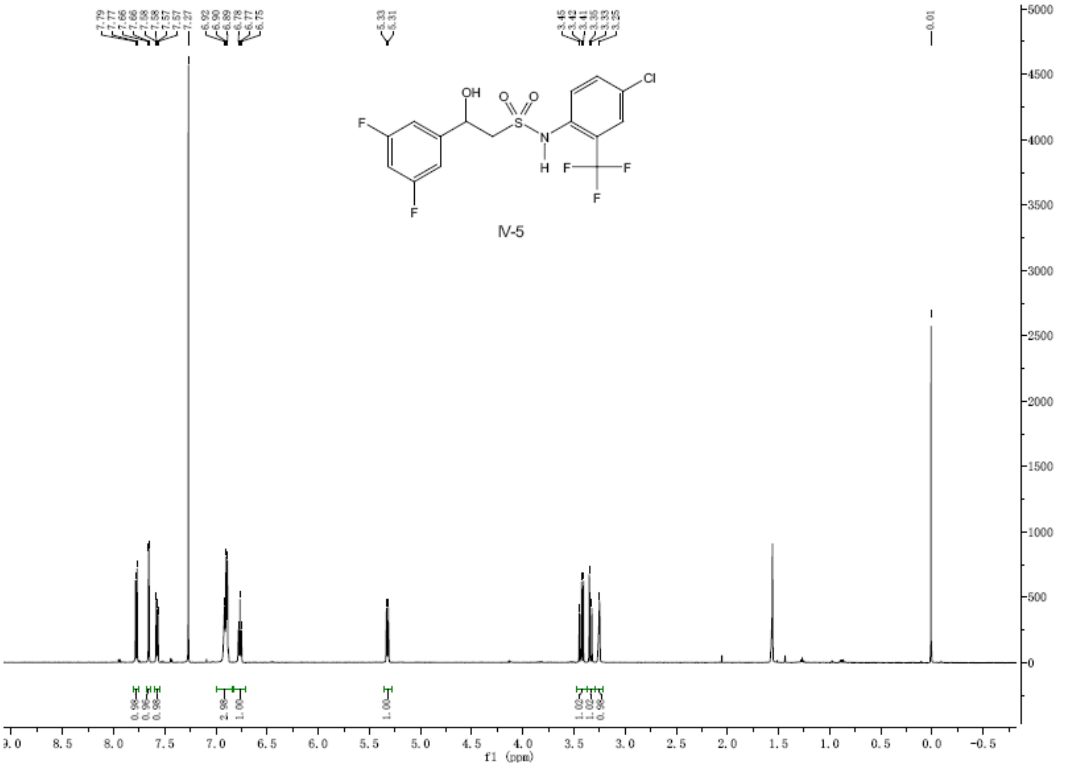
IV-5
f1 (ppm)

^{1}H-NMR spectra of title compounds **V1-V16**

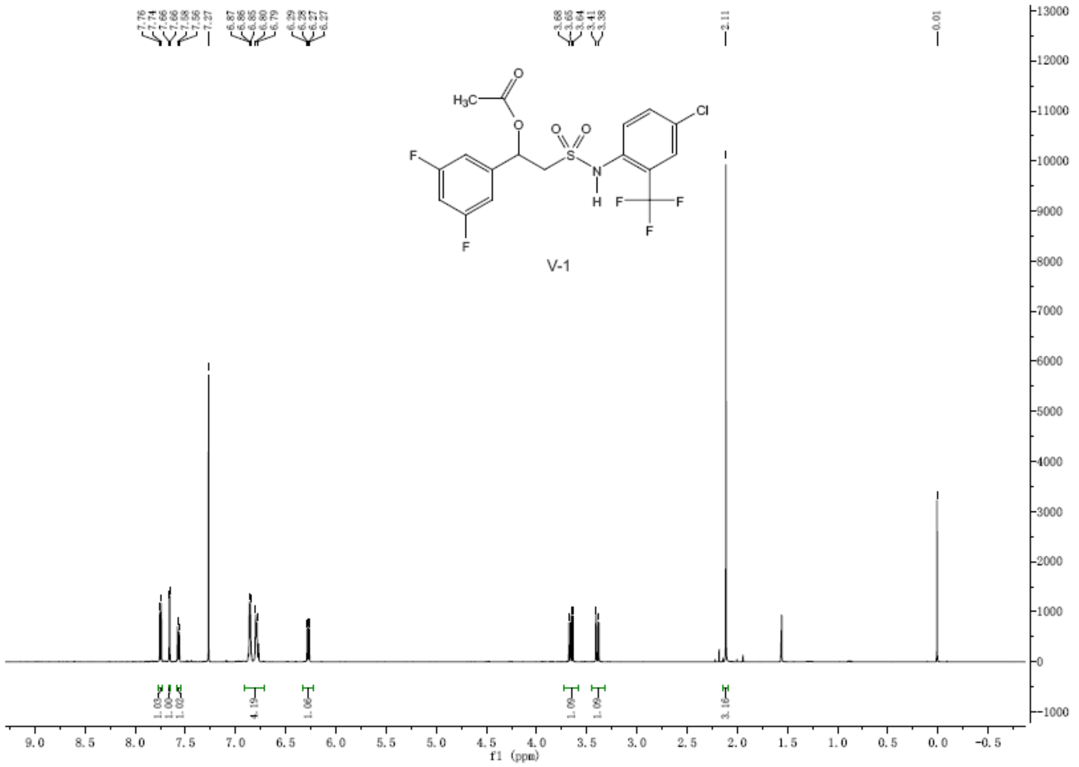

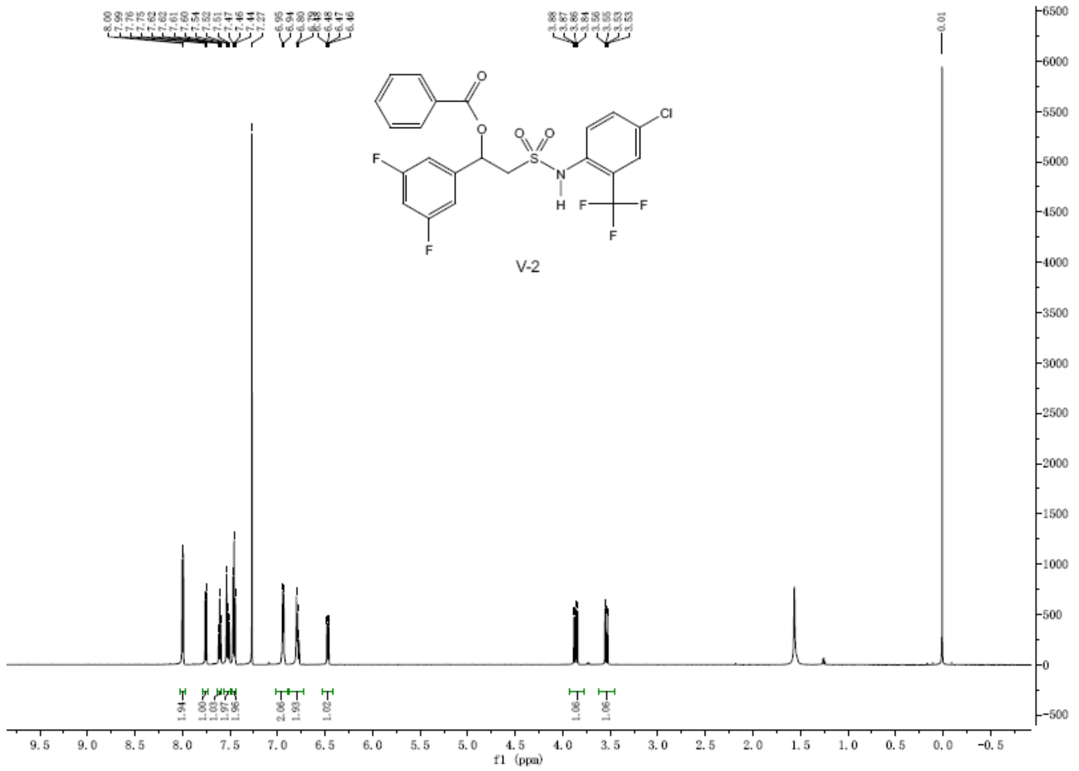

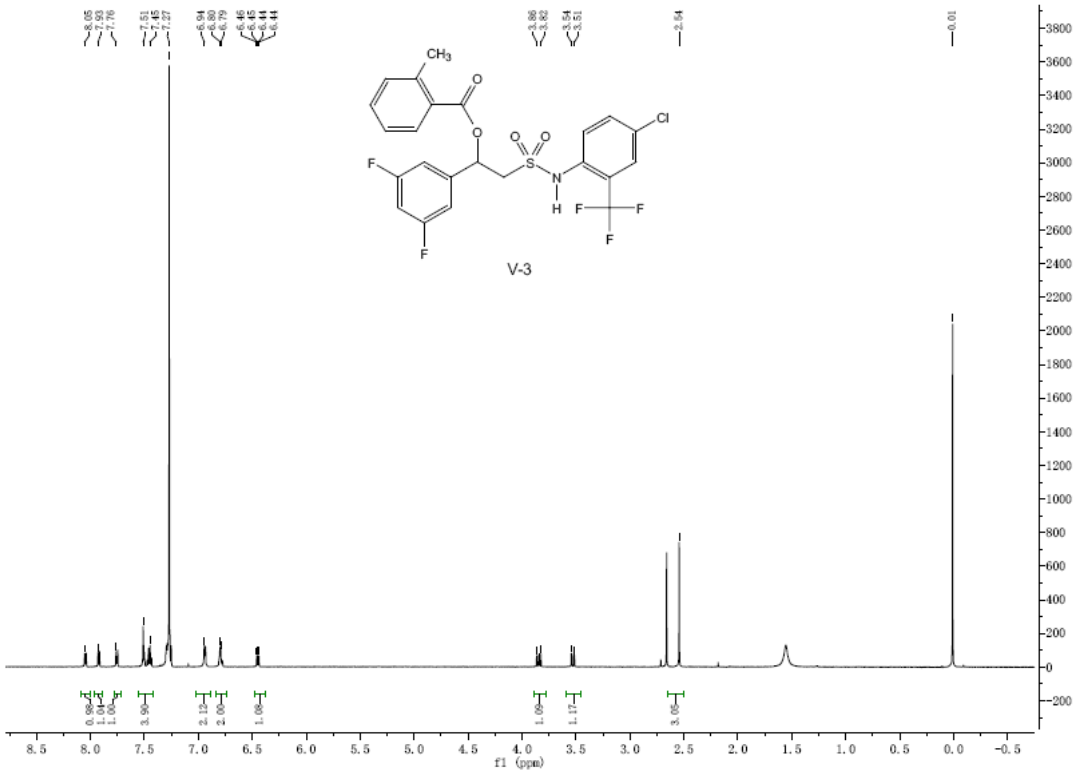
V-3
f1 (ppm)

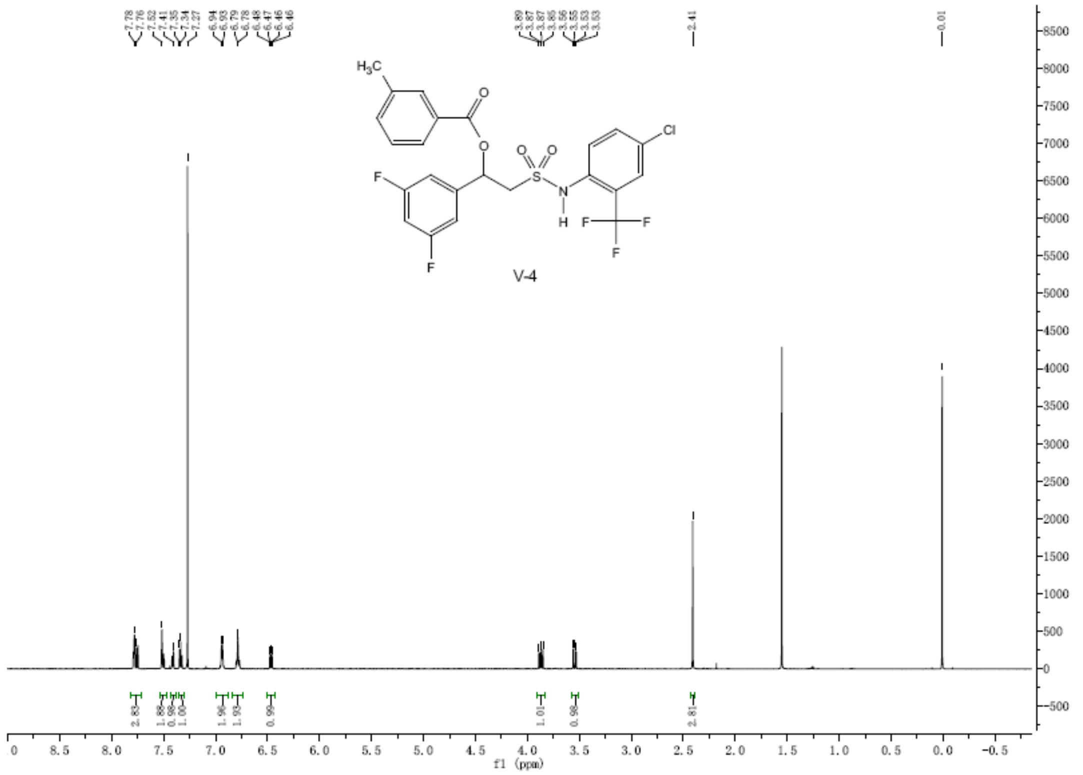
V-4
f1 (ppm)

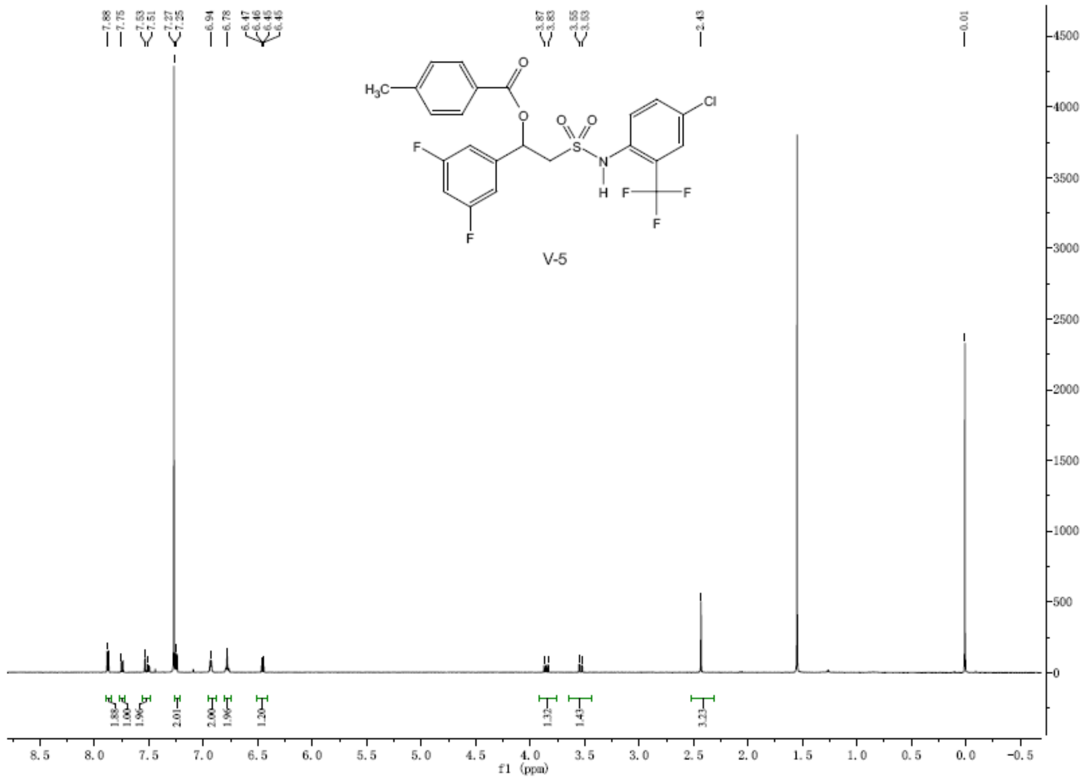
V-5
f1 (ppm)

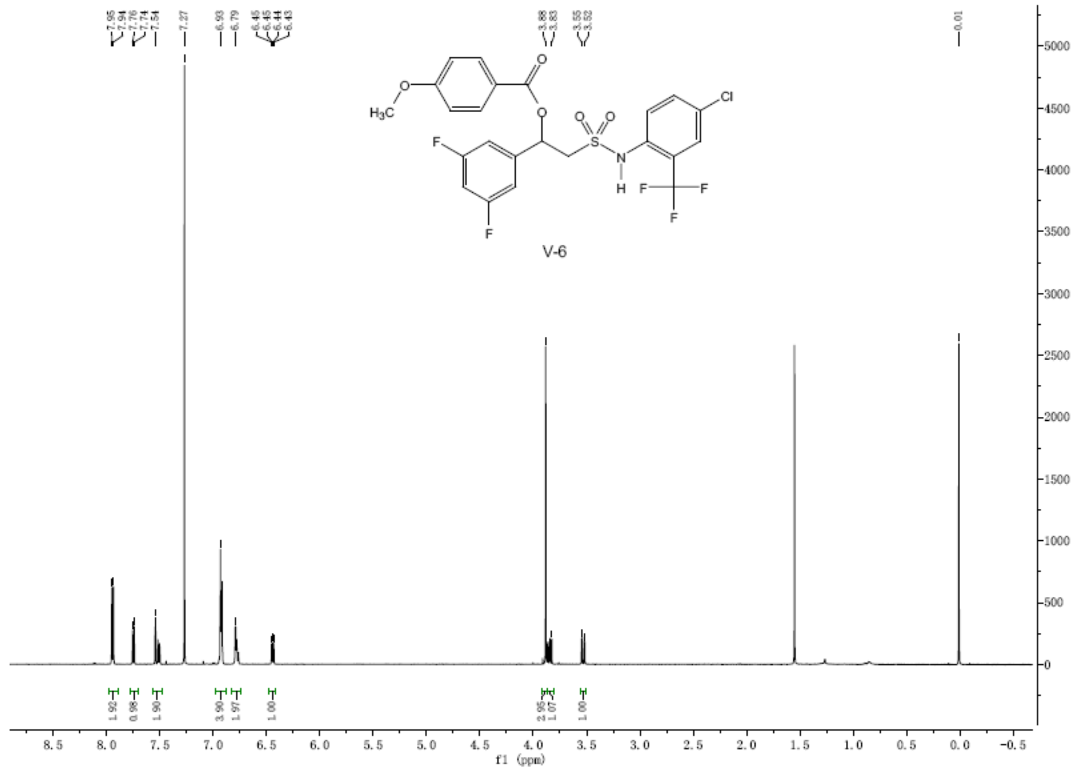
V-6
f1 (ppm)

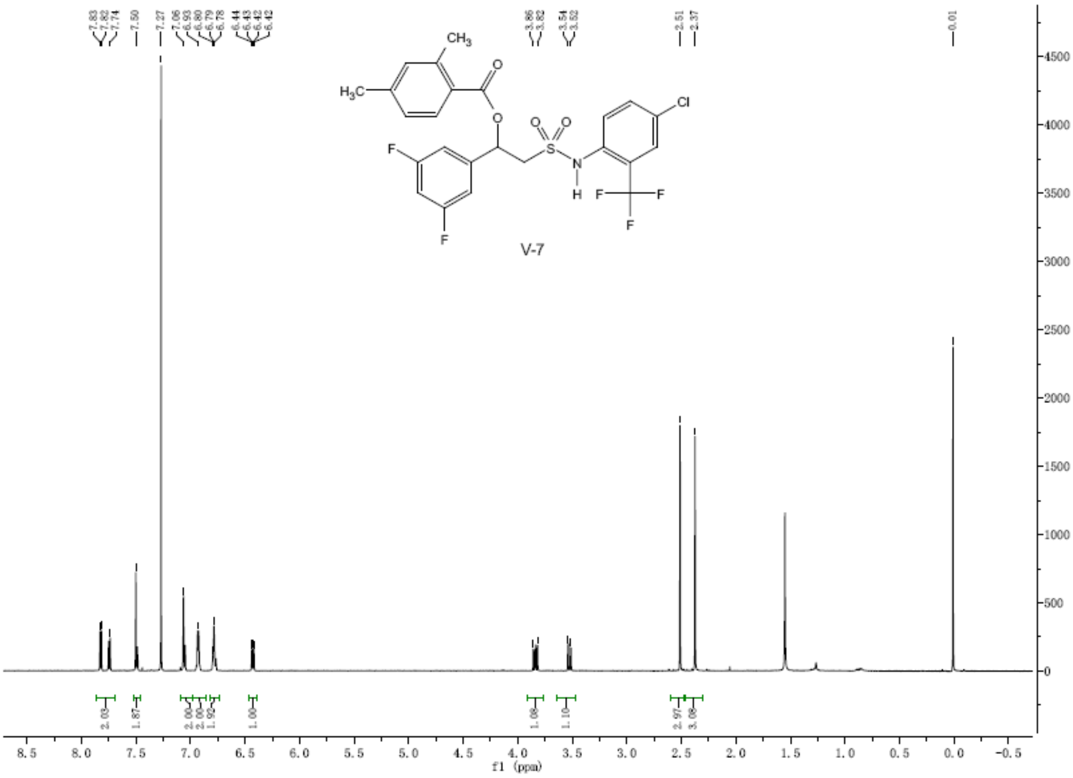
V-7
f1 (ppm)

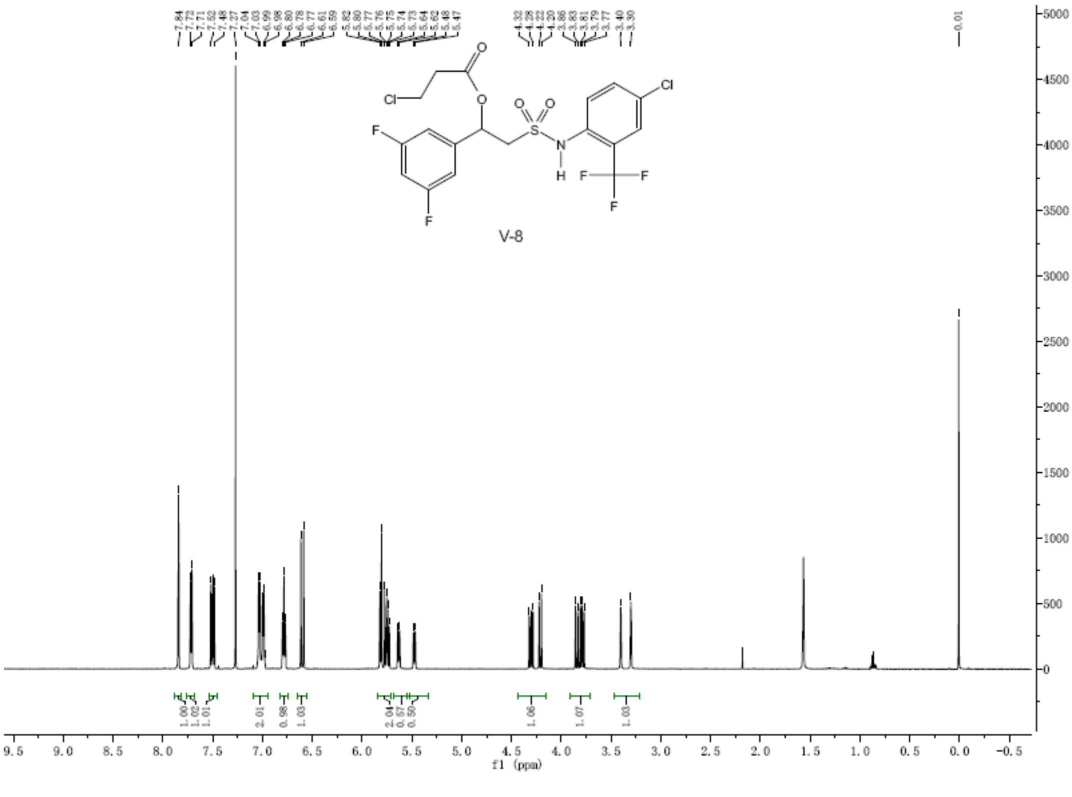
V-8
f1 (ppm)

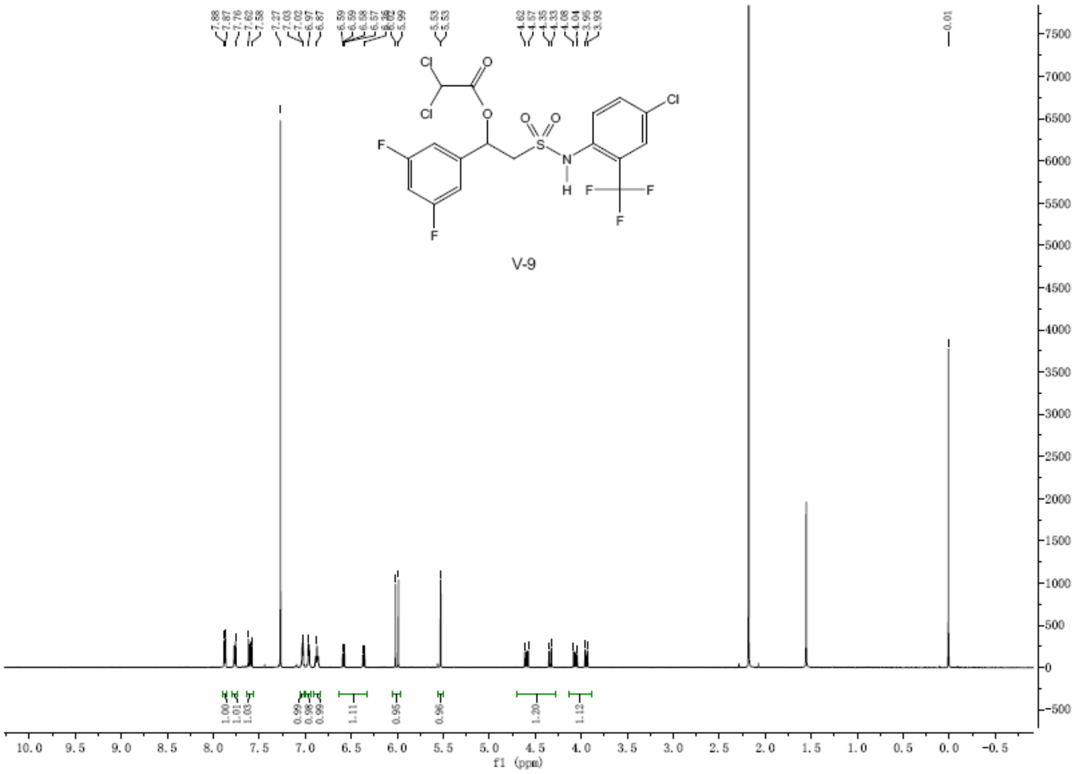

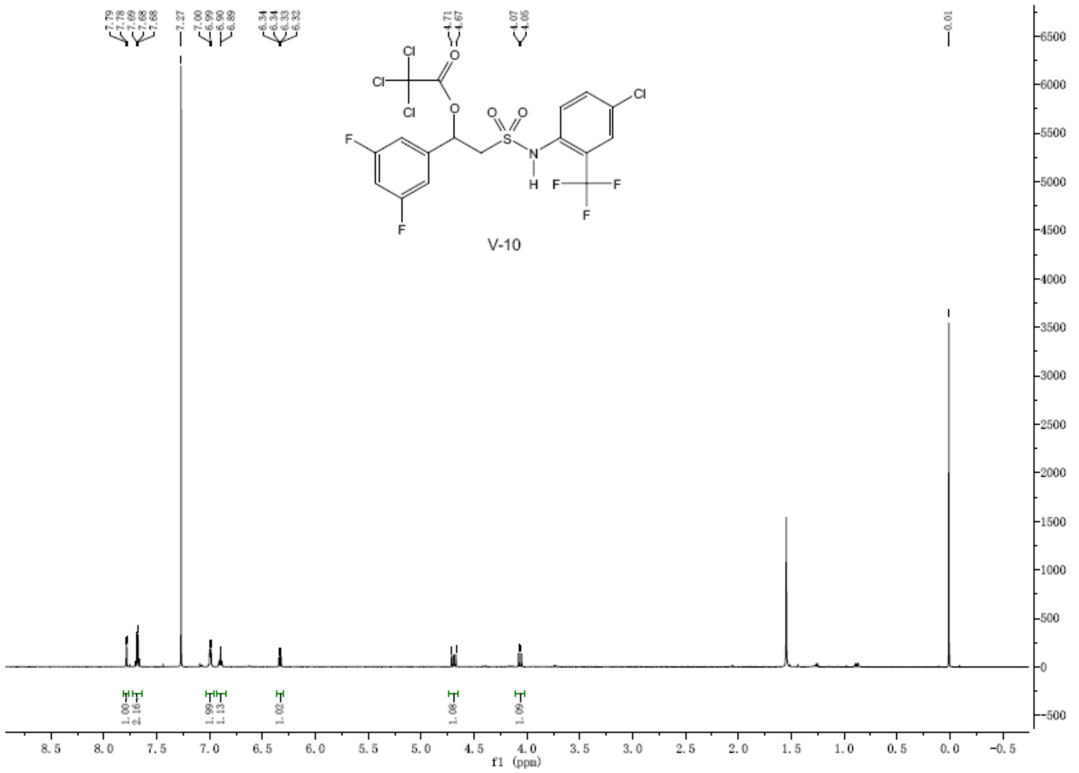

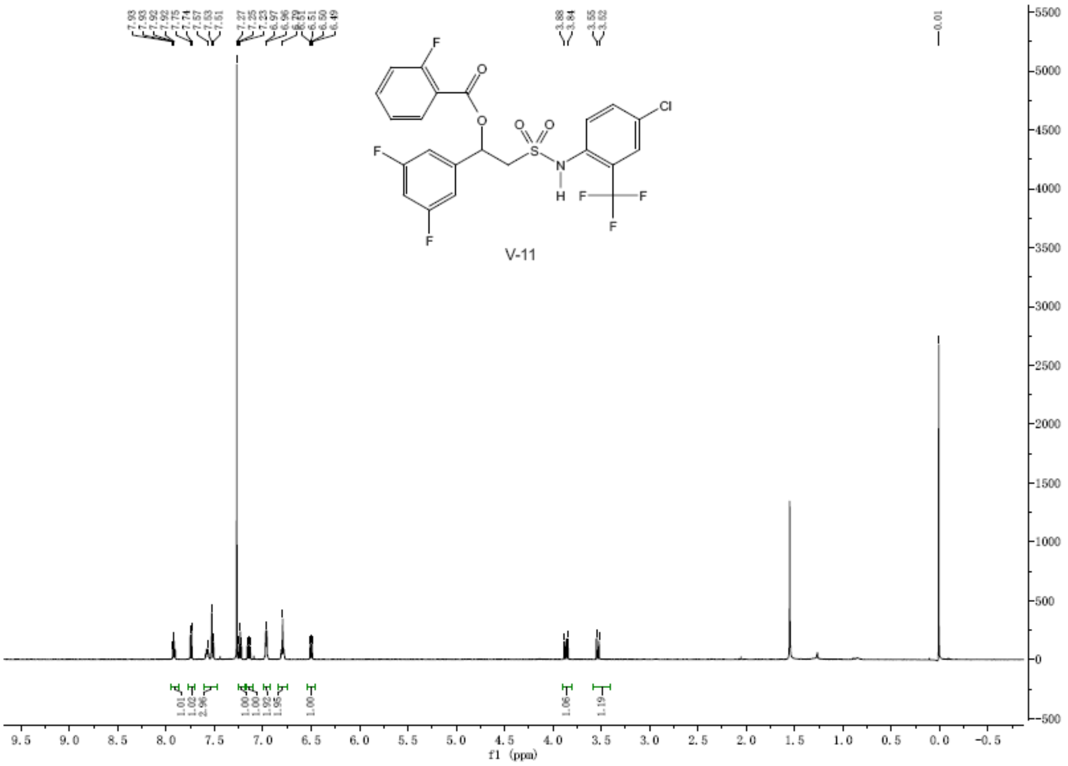
V-11
f1 (ppm)

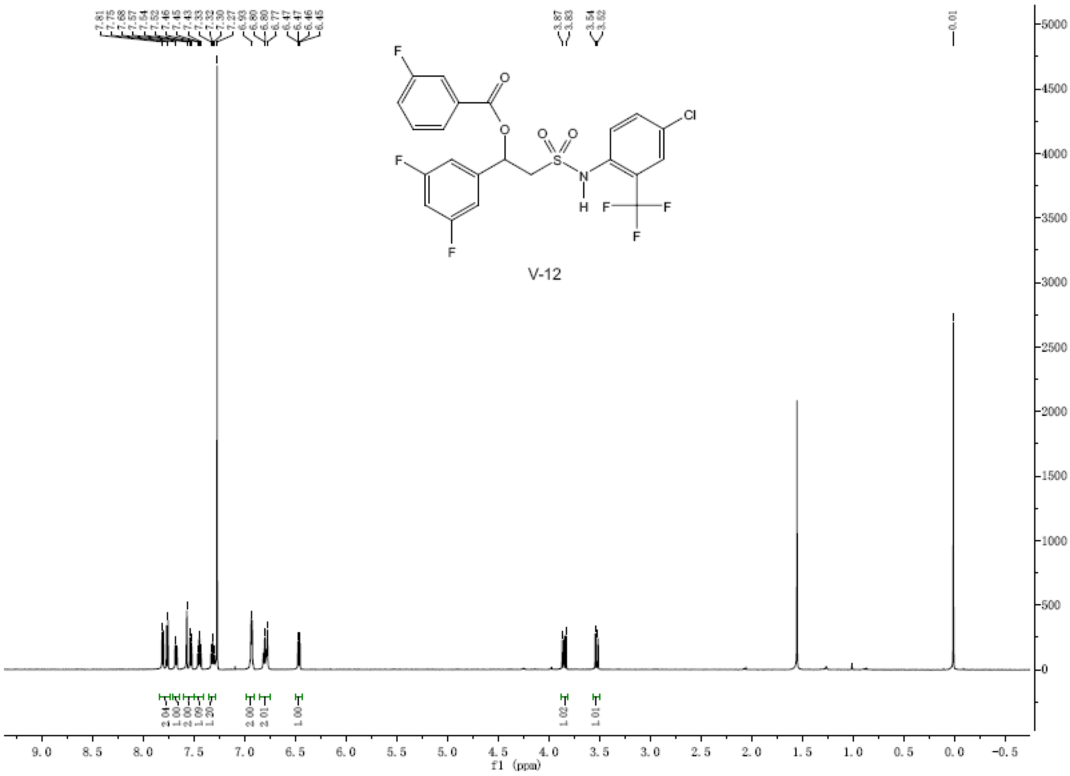
V-12
f1 (ppm)

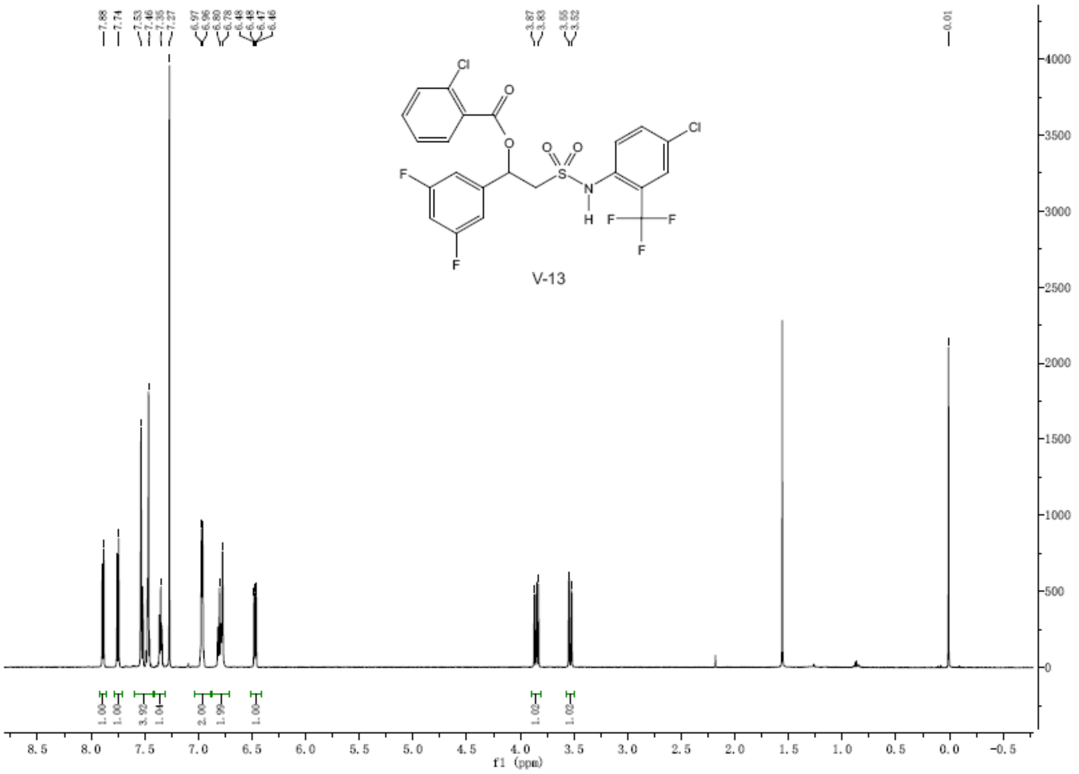

V-13

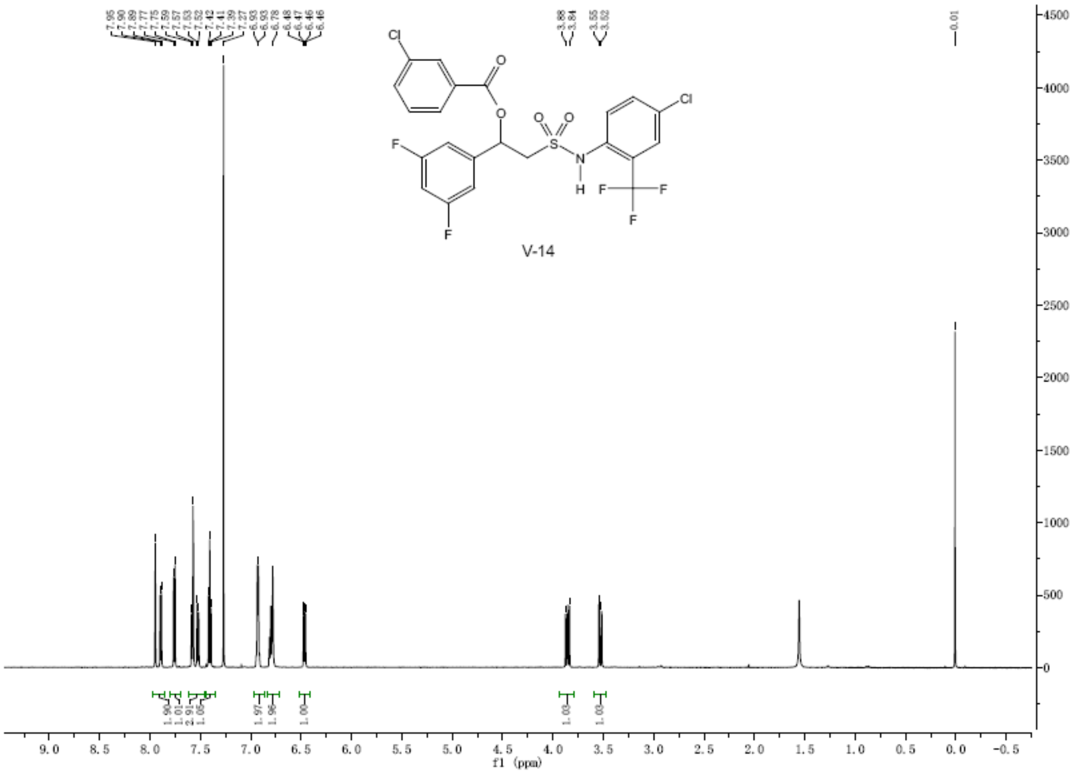

V-14

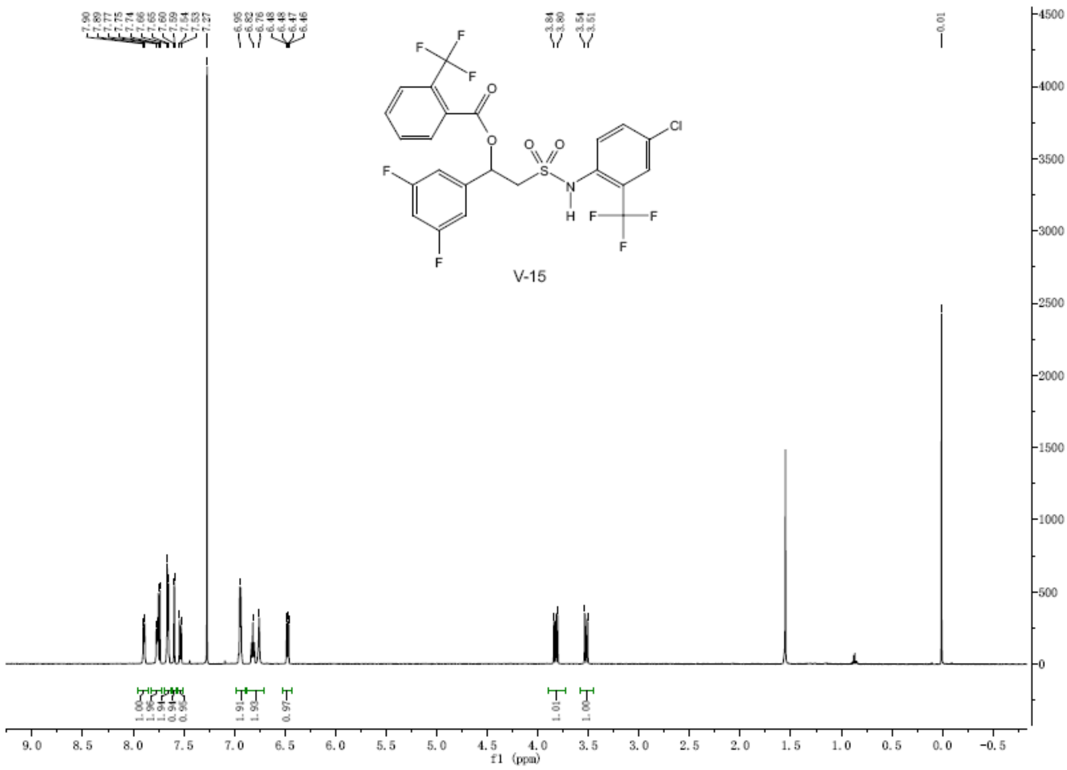
V-15
f1 (ppm)

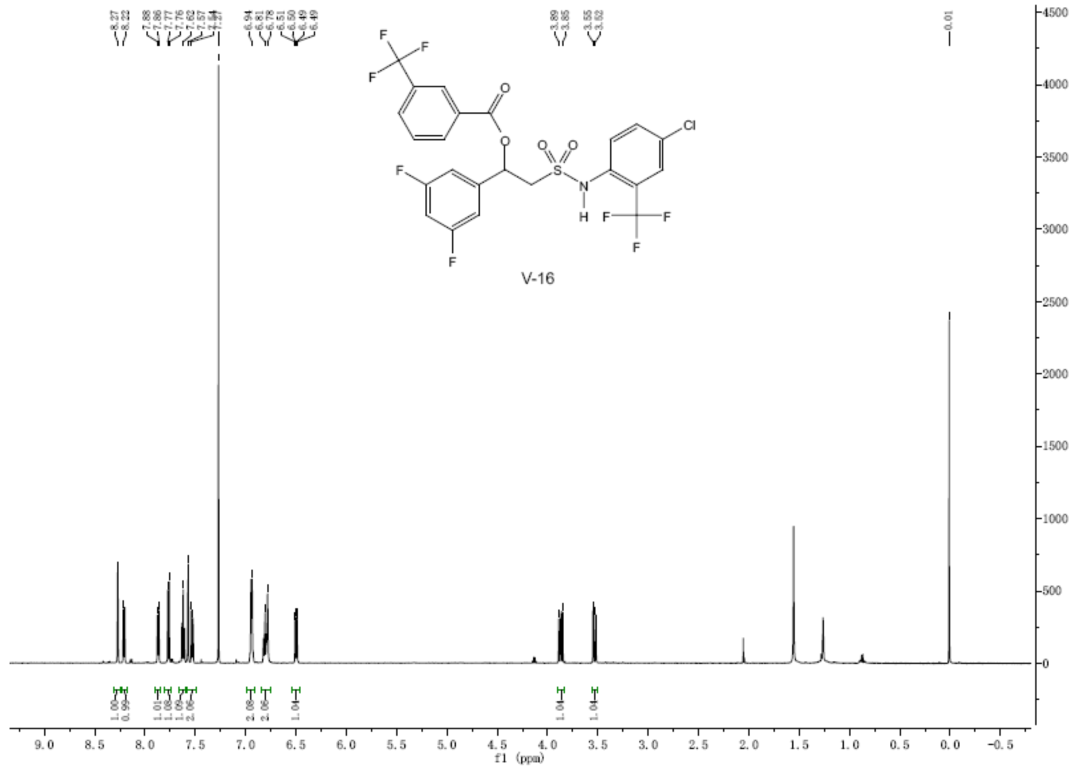
V-16
f1 (ppm)
